# Sox21 Regulates Anapc10 Expression and Determines the Fate of Ectodermal Organ

**DOI:** 10.1016/j.isci.2020.101329

**Published:** 2020-06-30

**Authors:** Kan Saito, Frederic Michon, Aya Yamada, Hiroyuki Inuzuka, Satoko Yamaguchi, Emiko Fukumoto, Keigo Yoshizaki, Takashi Nakamura, Makiko Arakaki, Yuta Chiba, Masaki Ishikawa, Hideyuki Okano, Irma Thesleff, Satoshi Fukumoto

**Affiliations:** 1 Division of Pediatric Dentistry, Department of Oral Health and Development Sciences, Tohoku University Graduate School of Dentistry, Sendai 980-8575, Japan; 2 Developmental Biology Program, Institute of Biotechnology, University of Helsinki, 00014 Helsinki, Finland; 3 Institute for Neurosciences of Montpellier, Inserm U1051, University of Montpellier, 34295 Montpellier, France; 4 Center for Advanced Stem Cell and Regenerative Research, Tohoku University Graduate School of Dentistry, Sendai 980-8575, Japan; 5 Section of Orthodontics, Division of Oral Health, Growth and Development, Faculty of Dental Science, Kyushu University, Fukuoka 812-8582, Japan; 6 Division of Molecular Pharmacology and Cell Biophysics, Department of Oral Biology, Tohoku University Graduate School of Dentistry, Sendai 980-8575, Japan; 7 Division of Operative Dentistry, Department of Restorative Dentistry, Tohoku University Graduate School of Dentistry, Sendai 980-8575, Japan; 8 Department of Physiology, Keio University School of Medicine, 35 Shinanomachi, Shinjuku-ku, Tokyo 160-8582, Japan; 9 Section of Pediatric Dentistry, Division of Oral Health, Growth and Development, Faculty of Dental Science, Kyushu University, Fukuoka 812-8582, Japan

**Keywords:** Rodent Dentistry, Developmental Genetics, Transcriptomics

## Abstract

The transcription factor Sox21 is expressed in the epithelium of developing teeth. The present study aimed to determine the role of Sox21 in tooth development. We found that disruption of Sox21 caused severe enamel hypoplasia, regional osteoporosis, and ectopic hair formation in the gingiva in Sox21 knockout incisors. Differentiation markers were lost in ameloblasts, which formed hair follicles expressing hair keratins. Molecular analysis and chromatin immunoprecipitation sequencing indicated that Sox21 regulated Anapc10, which recognizes substrates for ubiquitination-mediated degradation, and determined dental-epithelial versus hair follicle cell fate. Disruption of either Sox21 or Anapc10 induced Smad3 expression, accelerated TGF-β1-induced promotion of epithelial-to-mesenchymal transition (EMT), and resulted in E-cadherin degradation via Skp2. We conclude that Sox21 disruption in the dental epithelium leads to the formation of a unique microenvironment promoting hair formation and that Sox21 controls dental epithelial differentiation and enamel formation by inhibiting EMT via Anapc10.

## Highlights

• Sox21 was induced by Shh in dental epithelial cells
• Sox21 deficiency in dental epithelium caused differentiation into hair cells
• Sox21 deficiency did not cause differentiation into mature ameloblasts
• Anapc10 induced by Sox21 bound to Fzr1 and regulated EMT via Skp2

## Graphical Abstract

**Figure:**
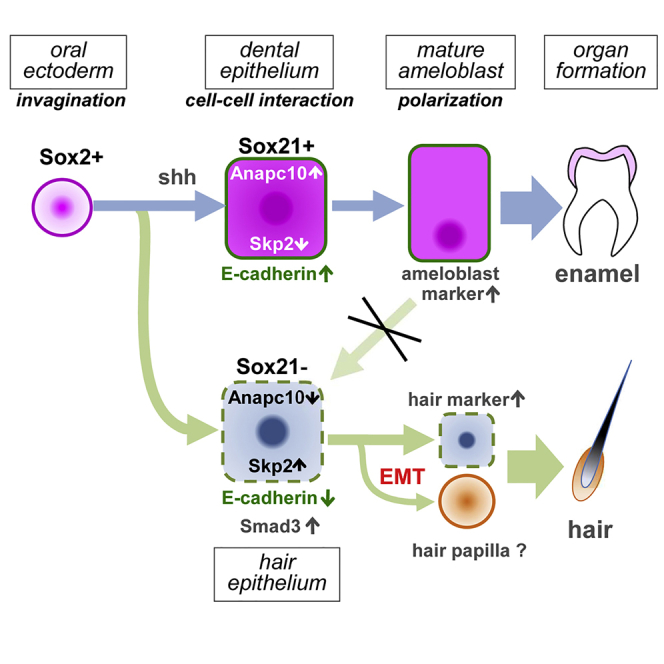

## Introduction

Members of the SRY-Box (Sox) B group share a conserved eight-amino-acid group B homology domain located adjacent to the HMG domain. The SoxB1 (Sox1–3) proteins function primarily as activators, whereas the closely related SoxB2 proteins (Sox14 and Sox21) are transcriptional repressors (Argenton et al., 2004; Sandberg et al., 2005). Furthermore, Sox21 was identified through proteomics studies to be associated with Sox2 during embryonic stem cell differentiation (Mallanna et al., 2010). The transcriptional activation/repression balance is important during the development of specific tissues. For example, SoxB2 repression of SoxB1 expression was shown to promote neural differentiation in the central nervous system (CNS) and peripheral nervous system (Sandberg et al., 2005; Uchikawa et al., 1999). This repression is supported by the expression of *Sox21* throughout the developing CNS and brain (Cunningham et al., 2008). In addition, a major role of Sox21 has been demonstrated during hair shaft cuticle differentiation (Kiso et al., 2009) and its deletion affects the hair lipid composition (Kawaminami et al., 2012). However, the SoxB1 group proteins and their roles have received greater attention to date (Donner et al., 2007; Driskell et al., 2009; Groves and Bronner-Fraser, 2000) than SoxB2 group involvement in developmental processes.

The development of most ectodermal organs is initiated from epithelial thickenings called placodes, and their morphogenesis involves invagination and folding of the epithelium regulated by reciprocal interactions between the mesenchyme and epithelium (Dhouailly, 2009). The cross talk between both tissues involves specific molecular signals, such as Wnt, bone morphogenetic protein (BMP), sonic hedgehog (Shh), Fgf, Eda, and Tgf (Jernvall and Thesleff, 2012; Liu et al., 2016; Miyazaki et al., 2016). The process of ectodermal organ morphogenesis is highly conserved and largely regulated by the same genes, hence various developmental defects are often observed concordantly in several ectodermal organs. For example, patients with syndromes such as incontinentia pigmenti (Smahi et al., 2000), Langer-Giedion (Momeni et al., 2000), Ellis-van Creveld (Ruiz-Perez et al., 2003), tricho-dento-osseous (Price et al., 1998), anhidrotic ectodermal dysplasia (Srivastava et al., 1996; van der Hout et al., 2008), hidrotic ectodermal dysplasia (Han et al., 2018; Lamartine et al., 2000), Hallermann-Streiff (Pizzuti et al., 2004), and Menkes (Tumer et al., 2003) have dysplasia in both teeth and hair.

The continuously growing rodent incisor represents a useful model to study stem cell regulation and organ development. Dental epithelial stem cells are localized in the proximal end of the incisor, and they express Sox2 and the Wnt inhibitor, Sfrp5 (Juuri et al., 2012). Dental epithelial cells differentiate into four types of epithelia: inner enamel epithelium (EE) and outer EE, stratum intermedium, and stellate reticulum. Inner EE expresses Shh, complementarily to Sfrp5, and differentiates into enamel-forming ameloblasts that express enamel matrix proteins, including amelogenin (Amel), enamelin (Enam), and ameloblastin (Ambn). Disruption of Amel or Ambn led to severe enamel hypoplasia, whereas hair abnormalities were not observed (Fukumoto et al., 2004; Gibson et al., 2001), indicating that these enamel matrix molecules are important for dental epithelium differentiation and enamel formation but not for hair development. Ameloblastin is critical for ameloblast differentiation in induced pluripotent stem cell-induced dental epithelium (Arakaki et al., 2012). In hair, the invaginated skin epithelium differentiates into interfollicular epidermis and hair follicles. After birth, adult stem cells residing in the basal layer of the epidermis and in the hair follicle bulge continuously regenerate the epidermis and hair follicles. Hair follicle stem cells derive from the bulge and migrate from the outer to the inner root sheath, where they express Keratin (Krt) 1, Krt10, Krt15, and Krt23 as epidermal keratins (Jensen et al., 1991; Rogers et al., 2004), as well as Krt27 and Krt32 as hair keratins (Langbein et al., 2010).

The present study focused on the role of Sox21 in tooth development. Although deletion of Sox21 is known to induce hair defects in mice (Kiso et al., 2009), deletion of the chromosome region 13q (containing the *SOX21* gene) in humans leads to irregular/dysplastic teeth (Kirchhoff et al., 2009).

## Results

### Sox21 Is an Ameloblast Marker Regulated by Shh

**Figure 1:**
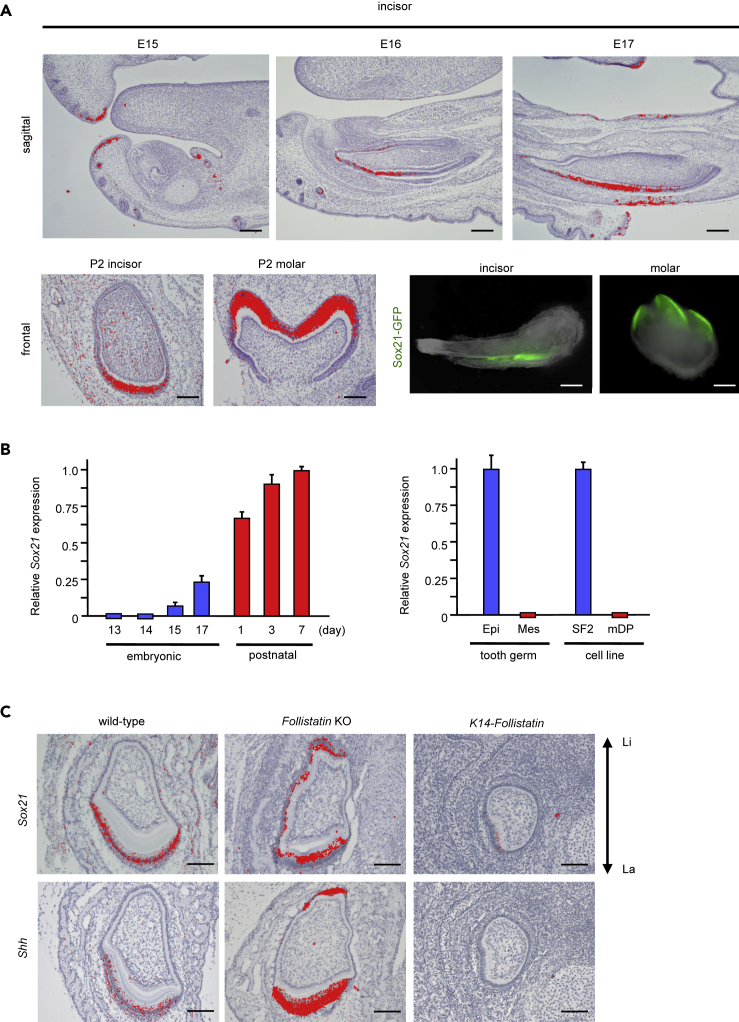
Expression of *Sox21* in the Development of Incisor and Molar Processes (A) Radioactive *in situ* hybridization (RISH) to analyze *Sox21* expression in mice at embryonic day (E) 15, 16, and 17 and postnatal day (P) 2. Upper panels, sagittal sections of incisors; lower left panels, frontal sections of incisor and molar; lower right panels, GFP expression in dissected incisors and molars; fluorescence is from *Sox21*-GFP in *Sox21*+/− P2 mice. Scale bar, 200 μm. (B) *Sox21* expression analyzed by qPCR. Tooth germs of each stage from E13 to P7 were collected (left panel). Dental epithelial cells and dental mesenchyme cells were isolated from the P1 tooth germ. In addition, SF2 cells (ameloblast cell line) and mDP cells (dental pulp cell line) were used for qPCR (right panel). Error bars represent mean ± SEM of five technical replicates. (C) *In situ* hybridization for the expression of *Sox21* and *Shh* in mutant mouse incisors. Scale bar, 200 μm. Li, lingual side; La, labial aspect of mandibular tooth.

The expression of *Sox21* mRNA during the tooth differentiation process was examined using *in situ* hybridization (Figure 1A). On embryonic day 15 (E15), *Sox21* was not detected in the dental tissue, but rather in part of the lip epithelium and in the whiskers. From E16 onward, *Sox21* expression was found in differentiating ameloblasts on the labial side of the incisor. *Sox21* expression was reinforced during ameloblast differentiation and at postnatal day 2 (P2); strong *Sox21* expression was detected in differentiated ameloblasts in the incisor and the molar (Figure 1A). To validate our findings, we used a reporter mouse carrying a GFP knockin at the *Sox21* locus (Kiso et al., 2009). As expected, GFP was detected in areas harboring differentiated ameloblasts in the mouse incisor and molar, i.e., the incisor labial side and the molar crown, respectively (Figure 1A). To evaluate the dynamics and specificity of *Sox21* expression, we monitored the expression by qPCR during tooth development and postnatal maturation. Although *Sox21* was not expressed at E13 and E14, its expression gradually increased between E15 and P7, reflecting the cell commitment and differentiation during tooth morphogenesis (Figure 1B). Furthermore, only epithelial cells isolated from the P1 incisor expressed *Sox21*, similar to the differential expression in the SF2 ameloblast cell line when compared with a mouse mesenchymal dental pulp cell line (Figure 1B).

As previously reported, Shh represents a marker of ameloblast differentiation (Iseki et al., 1996). Therefore, we analyzed *Shh* and *Sox21* expression in mutants exhibiting abnormal ameloblast differentiation. As expected, the modification of the region of ameloblast differentiation was reflected through *Shh* and *Sox21* expression. Follistatin is a BMP antagonist that regulates ameloblast differentiation. We used *Follistatin* knockout (KO) and *K14-Follistatin* transgenic mice to confirm whether Sox21 is a marker of ameloblasts. Specifically, ectopic ameloblast differentiation in the lingual side of the incisor, as observed in *Follistatin* KO mice (Saville, 1970), was correlated with the expansion of *Shh* and *Sox21* (Figure 1C). Similarly, the loss of ameloblast differentiation in *K14-Follistatin* mice (Wang et al., 2004) was associated with a lack of *Shh* and *Sox21* expression in the labial side (Figure 1C). Taken together, our results indicated that *Sox21* specifically marks the ameloblast lineage during terminal cell differentiation.

Ameloblasts are constantly renewed in the ever-growing mouse incisor. Their maturation begins at the cervical loop (CL), which is located posteriorly, and continues while the cells migrate anteriorly in the EE. To investigate the possible association between *Sox21* expression and Shh signaling, we first investigated the expression patterns of Shh signaling pathway genes in the mouse incisor (Figure S1A). Although *Sox21* expression was initiated only in differentiated ameloblasts, the Shh pathway genes *Shh*, *Gli1*, *Gli2*, and *Ptc2* were expressed in the CL and early EE. These results indicate that Shh activity preceded *Sox21* expression during ameloblast differentiation. To examine the association between Shh activity and *Sox21* expression further, we cultured E16 mouse incisors with or without the Shh inhibitor, cyclopamine. Through *in situ* hybridization, we showed that the absence of Shh activity greatly reduced the area of *Sox21* expression (Figure S1B). We confirmed this result by qPCR, which demonstrated that recombinant Shh enhances *Sox21* expression (Figure S1C) as rapidly as 4 h after initiation of treatment. The addition of recombinant Shh in the culture medium enhanced *Sox21* expression, whereas the presence of cyclopamine drastically reduced *Sox21* expression in the E16 incisor and P2 dental epithelium (Figure S1C). Based on the rapid effect of Shh activity on the expression of *Sox21*, we suggest that the Shh pathway directly regulates *Sox21* expression during ameloblast differentiation.

### *Sox21*-Deficient Mice Display Signs of Amelogenesis Imperfecta

**Figure 2:**
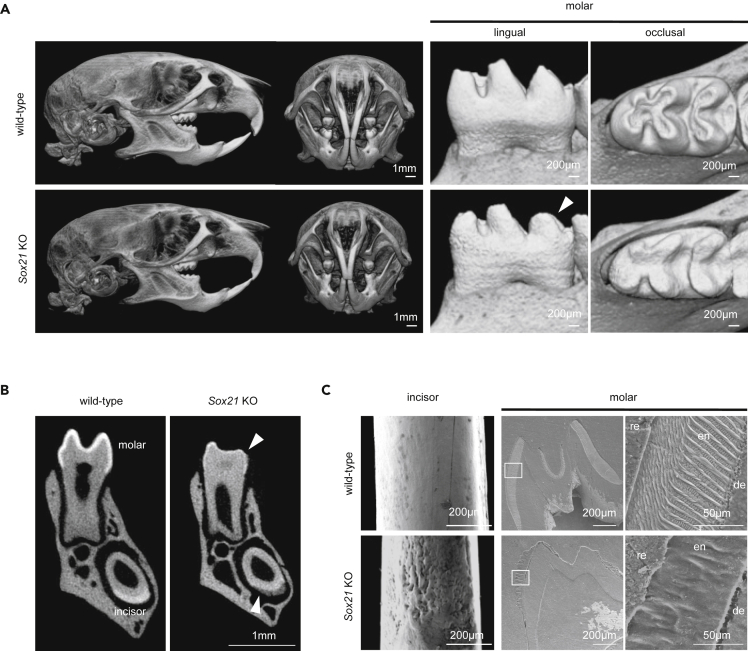
Phenotype of the Hard Tissue in *Sox21* KO Mouse (A–C) Wild-type and *Sox21* KO mice (6-week-old) as analyzed by micro-CT (A and B) and SEM (C) for tooth formation. (A) Stereo images of the whole head, skull, and tooth (molar). Arrowhead indicates the excessive dental attrition. (B) Tomogram of left mandibular tooth. Arrowhead indicates the enamel of the *Sox21* KO mouse. (C) SEM image showing tooth surface of the incisor (left panel). Low (middle panel) and high (right panel) magnification of a molar cross-section obtained by SEM. de, dentin; en, enamel; re, epoxy resin.

To understand the role of *Sox21* expression during ameloblast differentiation, we assessed the teeth of *Sox21* KO mice at 6 weeks of age, using micro-computed tomography (CT). In the head region, the size of the entire head remained unchanged and the molars exhibited excessive dental attrition in *Sox21* KO mice (Figure 2A). No morphological changes were observed in the dentin and roots of molars, but the surface of the enamel was seriously affected and rugged in both molars and incisors (Figures 2A and 2B). The enamel thickness in the molar crowns was markedly reduced (Figure 2B arrowhead). Scanning electron microscopy (SEM) showed a seriously rugged surface of the enamel in the incisors and the loss of enamel prism structure in the molars (Figure 2C). The evaluation of tooth calcification by micro-CT further indicated a drastic reduction in enamel density. *Sox21* KO incisors and molars showed only 9% and 6% of the enamel volume of the controls (Figure S2), although the dentin layer remained unaffected. Thus, we concluded that Sox21 is instrumental for proper enamel layer deposition.

### Sox21 Deficiency Leads to Loss of Ameloblast Polarity and a Change in Cell Fate toward Skin Identity

**Figure 3:**
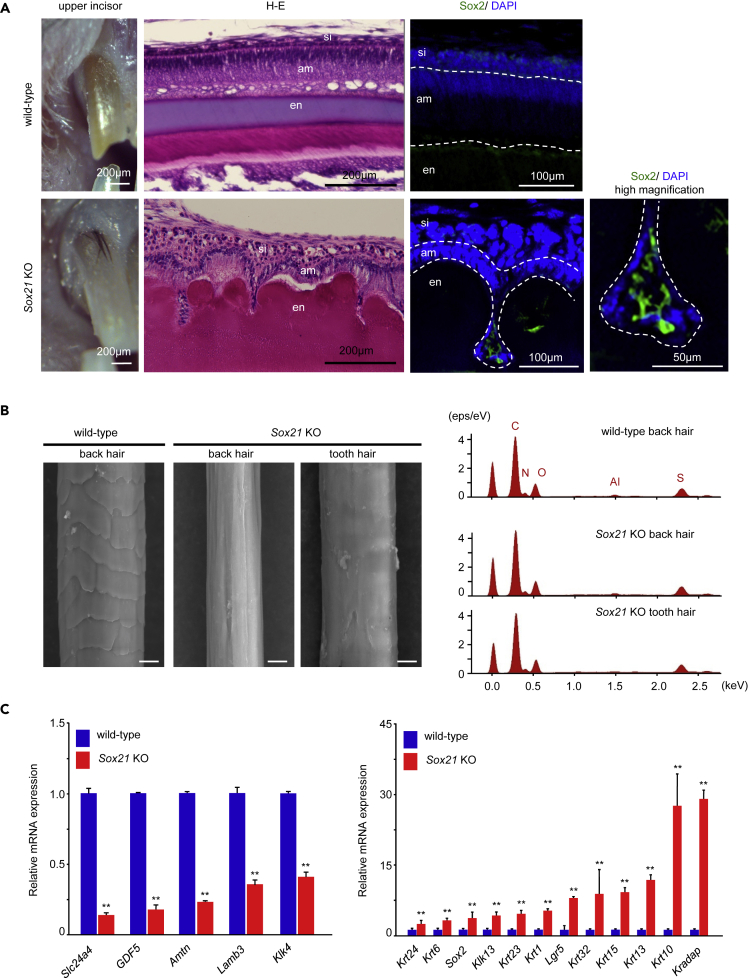
Histological Analysis of the *Sox21* KO Mouse Incisor (A) The upper incisors of 5-week-old wild-type and *Sox21* KO mice were observed using a stereo microscope (left panel). The lower incisors of a wild-type and a *Sox21* KO mouse were stained with hematoxylin and eosin (H&E) (middle panel). The right panels show high magnifications of ameloblasts in an incisor. The ameloblasts of the mouse incisor were immunostained using anti-Sox2 antibody with DAPI (right panel). si, stratum intermedium; am, ameloblasts; en, enamel. (B) Surface structure of a wild-type back hair, *Sox21* KO back hair, and *Sox21* KO tooth hair as imaged by SEM. Scale bar, 10 μm. The elemental mapping of the hair was analyzed by energy-dispersive X-ray spectrometry. (C) qPCR analysis of the expression of genes identified as being repressed or induced in the microarray data. P1 tooth germs of wild-type and *Sox21* KO mice were used for qPCR. Error bars represent mean ± SEM of five technical replicates. Student's t test (∗∗p < 0.001).

As *Sox21* is expressed in ameloblasts and loss of Sox21 leads to enamel defects, we analyzed the morphology of the ameloblast layer in 6-week-old *Sox21* KO mice. In wild-type mice, the ameloblasts formed a continuous epithelial monolayer of elongated polarized cells. The nuclei were at the cell base, and the enamel layer was deposited from the cell apex (Figure 3A). Upon Sox21 deficiency, the ameloblasts were poorly elongated, and some cells had lost their polarity. Some cells were embedded in the enamel layer, forming pits within the mineralized tissue (Figure 3A). Furthermore, the cells at the bottom of the pits expressed *Sox2*. Sox2 expression has been shown to mark dental epithelial stem cells in the cervical loop of the mouse incisor (Juuri et al., 2012); Sox2 is then markedly downregulated in differentiated ameloblasts (Figure S3). Cell proliferation was analyzed by 5-ethynyl-2’-deoxyuridine (EdU) staining. EdU assays measure the incorporation of EdU into newly synthesized DNA. Sox2 expression remained in the differentiated dental epithelium, such as ameloblasts (Figure S3 arrowhead), whereas expression of Sox2 did not affect cell proliferation because EdU was unchanged in *Sox21* KO mice (Figure S3). In the embryonic stage of incisor and molar development, no histological differences in tooth germ layers were observed between wild-type and *Sox21* KO mice (Figure S4A). Furthermore, the disorganization of the dental epithelium was observed on postnatal day 7 molars in *Sox21* KO mice (Figure S4B arrow). In the case of incisors, the dental epithelium from the embryonic stage to postnatal day 7 was normal (Figure S4C). However, disorganization and lost polarity were obtained in the anterior dental epithelium of P4-week-old incisors, and the epithelium invaginated and formed pits at 6 week (Figures S4D and S4E). Therefore, the expression of *Sox2* in the bottom of the enamel pits reflected dedifferentiation of the ameloblasts to remaining Sox2-positive dental epithelial stem cells.

Oral examination revealed that in 63% of 5- to 6-week-old *Sox21*-deficient mice, hairs appeared between the tooth surface and the gingival sulcus in both the lower and upper jaws (Figures 3A, 5A, and S5A), but not in the molars. Sox21 was not expressed in the early embryonic stage (Figures 1A and 1B), and the ameloblasts had normal morphology until P7 in *Sox21* KO mice incisors (Figures S4A and S4C). The cell arrangement of the ameloblast layer was disturbed in postnatal 4-week-old incisors (Figure S4D). However, ectopic hair was not formed in this stage. Hair was formed within the gingiva at 5 weeks and erupted at 5 to 6 weeks (Figure S5A). From this result, ectopic hair was formed in the secretory stage of the ameloblast layer at around 5 weeks, but not the early stage of tooth development. Ectopic hair contains medulla and cortex structure of the hair, as well as follicle papilla cells (Figure S5B). Such incomplete hair is observed with teratomas and dermoid cysts (Ahmad et al., 2013; Komiyama et al., 2002; Lewis et al., 2012; Takeda et al., 2003; Tavares et al., 2018). However, no typical bulge structure was observed, and we did not find any teratoma-like structure in *Sox21* KO incisors. Previously, it was reported that Sox21 deficiency leads to a modification of the hair cuticle layers, resulting in defective anchorage, and. ultimately, to cyclic alopecia (Kiso et al., 2009). Thus, we compared the surfaces of dental and back skin hairs. The surface of the back hairs was smooth in *Sox21* KO mice and different from that of the wild-type mice (Figure 3B). Notably, the tooth hairs in *Sox21* KO mice displayed a smooth surface, reflecting the lack of a cuticle, which was similar to the back hair in *Sox21* KO mice. Furthermore, analysis of the elemental mapping at the compositional and microstructural levels by energy-dispersive X-ray spectrometry revealed high similarity between the different hair types (Figure 3B). From macroscopic analyses, some back hairs appeared thin with zigzag shape, whereas the tooth hairs were short and straight (Figure S5C). The tooth hairs were thicker than the back hairs (Figures 3B and S5C). Therefore, the length of each hair was measured (Figure S5D). The average length of the tooth hairs was 2.49 ± 0.36 mm, whereas that of the back hairs was 3.19 ± 0.32 mm (p < 0.005, Student's t test). Tooth hairs were significantly shorter than back hairs.

To investigate whether ectopic hairs of *Sox21* KO mice switched tooth cell fate, microarray analysis were performed between tooth and skin in wild-type (Table S1). Conversely, almost all the genes upregulated in *Sox21* KO teeth were more highly expressed in the skin. Notably, numerous keratin-related genes were upregulated in the *Sox21* KO tooth germ and wild-type skin. The microarray data were validated by qPCR. Specifically, *Amtn* and *Klk4* were decreased in *Sox21* KO ameloblasts (Figure 3C), whereas the expression of *Shh*, *Gli1*, *Gli2*, *Ptc2*, *Mmp20*, and *Amel* remained unchanged (data not shown). As for the microarray results, the expression of dental epithelial stem cell marker *Sox2*, as well as keratin family and hair-related genes, such as *Lgr5*, were increased in *Sox21* KO dental epithelium (Figure 3C). Lgr5 is expressed in hair germ and hair bulge of the hair root sheath wall region (Leung et al., 2018), whereas Sox2 is expressed in the dermal papilla of the hair root sheath bottom (Driskell et al., 2009). The expression of Sox2 and Lgr5 was examined by immunostaining in 6-week-old mouse incisors (Figures 3A and S6A). These expression patterns were similar to that in the hair root sheath, as Sox2 was expressed in the bottom and Lgr5 was expressed in the wall of the ameloblast invagination pit. Taken together, these results reflected the perturbed differentiation of ameloblasts and a cell fate change from dental to skin identity in the *Sox21* KO incisors.

### *Anapc10* Is a Sox21 Direct Target Gene Involved in Ameloblast Differentiation

**Figure 4:**
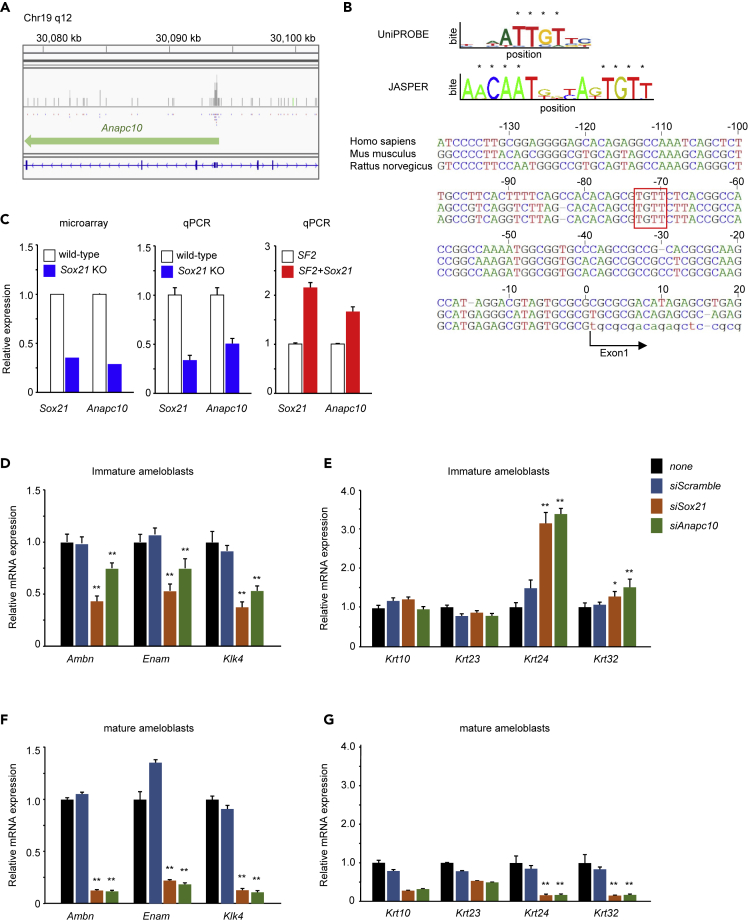
Identification of Sox21 Downstream Molecules and Its Effect on Ameloblasts (A) Chromatin immunoprecipitation sequencing analysis of Sox21 interactors in SF2 cells. The peak of bound chromatin fragments representing *Anapc10* was visualized using IGV software. (B) Sox21 binding sites were investigated in UniPROBE and JASPAR databases (top). The upstream of *Sox21* of *Homo sapiens*, *Mus musculus*, and *Rattus norvegicus* were aligned. Capital letters represent intronic nucleotides. (C) Expression levels of *Sox21* and *Anapc10* in the ameloblasts of *Sox21* KO and wild-type mice as investigated by microarray analysis (left) and qPCR (middle). Sox21 overexpression in SF2 cells as examined by qPCR (right). Error bars represent mean ± SEM of five technical replicates. (D–G) Expression of *Sox21* and *Anapc10* in SF2 cells was inhibited by siRNA. SF2 was cultured by NT-4 for 48 h to differentiate to mature ameloblasts (F and G). Scramble siRNA was used as a negative control. After 48 h, the expression of ameloblast markers (D and F) and keratin family genes (E and G) was evaluated by qPCR. Error bars represent mean ± SEM of five technical replicates. Student's t test (∗p < 0.005, ∗∗p < 0.001).

To determine the role of Sox21 in amelogenesis, we attempted to determine Sox21 direct target genes via chromatin immunoprecipitation sequencing analysis (Figure 4A). Among the direct targets identified, a Sox21 binding peak was detected in the promoter region of anaphase-promoting complex subunit 10 (*Anapc10*) (Figure 4A). Subsequently, a candidate Sox21-binding consensus sequence (Matsuda et al., 2012) was identified in the *Anapc10* promoter region, using the UniPROBE (http://thebrain.bwh.harvard.edu/uniprobe/) and JASPER (http://jaspar.genereg.net) databases (Figure 4B). qPCR validation of the effects of Sox21 deficiency or excess on the expression of these target genes indicated that only *Anapc10* expression was modulated by the Sox21 level (Figure 4C). These results indicate that the expression of *Anapc10* was dependent on Sox21 in ameloblasts. Anapc10 has previously been shown to regulate the cell cycle through cyclin B ubiquitination (Kominami et al., 1998). However, the role of Anapc10 in the dental context was unclear. Therefore, we examined the effects of Sox21 and Anapc10 on the expression of *Ambn*, *Enam*, and *Klk4*, which are markers of proach to detect molecular changes induced by Sox21 deficiency (Table S1). We dissected tooth germs from P1 *Sox21* KO and control incisors. In particular, several markers of ameloblast maturity (i.e., amelotin [Amtn], laminin α3 [Lama3], and kallikrein-related peptidase 4 [Klk4]) were included among the downregulated molecules in the *Sox21* KO tooth germ. Hair and tooth development-related genes were largely similar between the P1 incisor tooth germ, including epithelium and mesenchyme, and P1 skin, including hair follicles (Thesleff and Mikkola, 2002). Except for *Wnt16*, genes that were downregulated in the *Sox21* KO tooth germ were also differentiated ameloblasts in the rat dental epithelial cell line SF2. SF2 is one of the immature (undifferentiated) dental epithelial cell lines that differentiates into ameloblasts when stimulated with neurotrophin 4 (NT-4) *in vitro* (Yoshizaki et al., 2008). Upon knocking down either *Sox21* or *Anapc10*, immature and mature ameloblasts reduced ameloblast marker expression (Figures 4D and 4F). Furthermore, the expression of dermal keratin *Krt24* and hair keratin *Krt32* was increased following *Sox21* or *Anapc10* knockdown in immature ameloblasts (Figure 4E). In differentiated SF2 cells, after stimulation by NT-4 and knocking down either *Sox21* or *Anapc10*, no hair marker expression was observed (Figure 4G). Our results suggest a genetic network involving *Sox21* and *Anapc10* upstream of *Ambn*, *Enam*, and *Klk4* during the terminal differentiation of ameloblasts. Furthermore, immature ameloblasts (such as pre-ameloblasts), but not differentiated ameloblasts, may undergo *trans*-differentiation into hair cells from dental epithelium.

### Sox21 Disruption Enhances Epithelial-To-Mesenchymal Transition and TGF-β1 Signaling in Dental Epithelium

**Figure 5:**
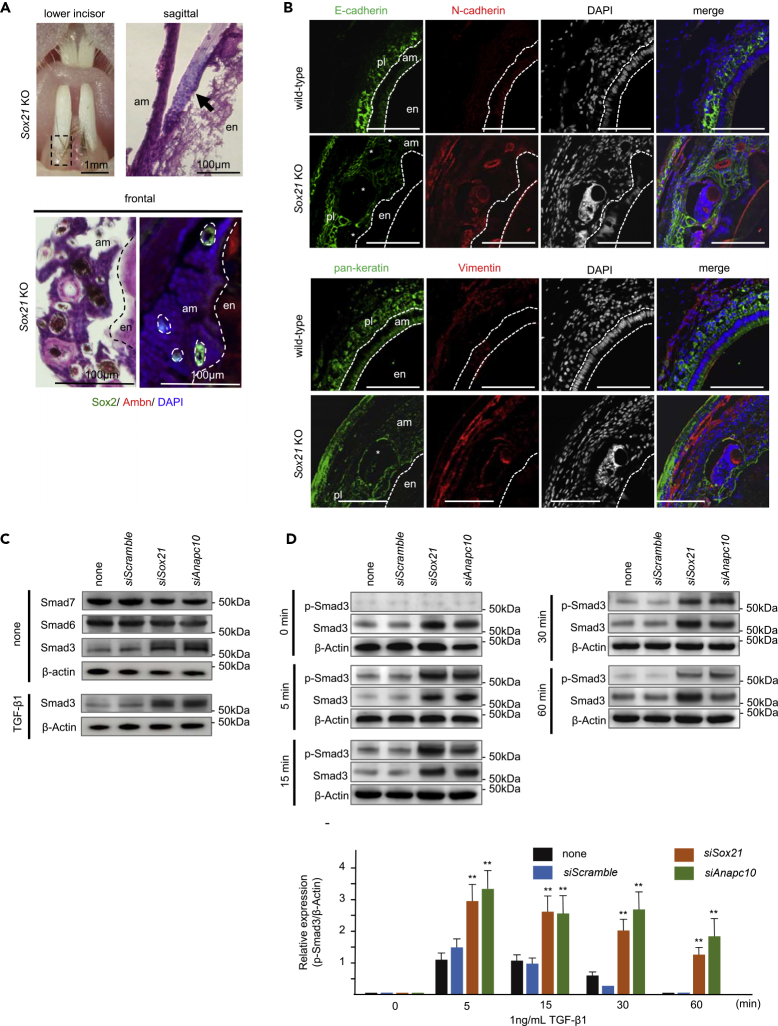
Molecular Analysis of the Ameloblasts in the *Sox21* KO Mouse (A) Histological analysis of the *Sox21* KO mouse lower incisor gingiva. H&E staining of sagittal and frontal sections of the hair shaft in ameloblasts and immunostaining of Sox2 and Ambn in the frontal section. am, ameloblast; en, enamel. (B) Double immunostaining of incisal frontal section from a 6-week-old mouse, counterstained with DAPI. Upper panels show sections stained using anti-E-cadherin and anti-N-cadherin antibodies; for lower panels, we utilized anti-pan-keratin and anti-Vimentin. Scale bar, 200 μm. am, ameloblast; en, enamel; pl, papillary layer. ∗root sheath analog. (C and D) Expression of *Sox21* and *Anapc10* in SF2 cells was inhibited by siRNA for 48 h (C) Smad3, 6, and 7 protein levels as detected by western blotting after SF2 cells were cultured with or without TGF-β1 (1 ng/mL) for 24 h. (D) *Sox21-* or *Anapc10-*silenced SF2 cells were stimulated with TGF-β1. After 5, 15, 30, or 60 min, Smad3 and p-Smad3 expression was analyzed by western blotting. The p-Smad3 expression was normalized to that of β-actin. The amount of p-Smad3 in control SF2 cells induced by TGF-β1 after 5 min was calculated as a standard. Relative expression of p-Smad3 is indicated. Error bars represent mean ± SEM of three technical replicates. Student's t test (∗∗p < 0.001).

We next examined the cells forming the ectopic tooth hairs. The histological analysis of the tooth hair showed presence of a hair shaft and a population of cells at the hair root (Figures 5A and S5B). As the hair follicle structure was not clear on sagittal sections, we used frontal sections to visualize this structure. Notably, we detected Sox2 expression in the hair root sheath localized in the enamel pits, whereas ameloblastin was absent from the hair structures (Figure 5A).

In the tooth hair, morphogenesis relies on cross talk between the epithelial and mesenchymal compartments. The development of ectopic tooth hairs upon Sox21 deficiency implies the presence of a dermal papilla at the base of the hair. To investigate this, we analyzed the presence of dermal markers in the bottom of the enamel pits. Expression of Sox2, which is known to be a dermal papilla marker (Driskell et al., 2009), was detected in the pits (Figures 3A and 5A). To examine this further, we assessed the presence of additional dermal papilla markers, N-cadherin and Vimentin. Although these markers were only weakly expressed in differentiated ameloblasts, loss of Sox21 induced strong expression of both cadherins in the ameloblast layer (Figure 5B). Furthermore, N-cadherin was strongly expressed in the root sheath of the *Sox21* KO tooth hairs. Similarly, pan-keratin was detected in enamel matrix-secreting mature ameloblasts and in the papillary layer, and Sox21 deficiency induced strong N-cadherin and Vimentin expression in the tooth hair root sheath (Figures 5B and S6B arrow). As our results suggested the appearance of dermal cells at the base of the tooth hair, we subsequently investigated the impact of *Sox21* and *Anapc10* knockdown on mesenchyme markers, such as *N-cadherin*, *Vimentin*, *Zeb1*, *Zeb2,* and *Snail1,* in the SF2 cell line (Figures S7A and S7B). As anticipated, the mesenchymal markers were all upregulated upon *Sox21* or *Anapc10* silencing in only immature ameloblasts. Our results therefore support the roles of Sox21 and Anapc10 in the maintenance of epithelial identity; when one of these are lacking, the differentiating ameloblasts can initiate epithelial-to-mesenchymal transition (EMT).

EMT is primarily induced through Smad3 and 4, which are under the control of Tgf activity (Kaimori et al., 2007; Zavadil et al., 2004). Therefore, we used small interfering RNAs (siRNAs) in SF2 cells to suppress the levels of Sox21 and Anapc10 and analyze the impact on Smad3, Smad6, and Smad7 by western blotting. The silencing of *Sox21* and *Anapc10* did not affect the Smad 6 and 7 levels in the cells; however, the amount of Smad3 increased drastically (Figure 5C). Notably, these results were transforming growth factor (TGF)-β independent, because the increase in Smad3 did not change with or without TGF-β1. To monitor the effects of *Sox21* and *Anapc10* silencing on TGF-β activity, we measured the kinetics of Smad3 phosphorylation upon TGF-β activation (Figure 5D). The effect of TGF-β1 on the SF2 cells, visualized by the presence of phospho-Smad3, was greatly enhanced by *Sox21* and *Anapc10* knockdown as rapidly as 5 min after TGF-β1 addition. These results suggest that the EMT process seen in *Sox21* KO ameloblasts originated from TGF-β activity through the phosphorylation of Smad3.

### Anapc10 and Fzr1 Regulate EMT by Modulation of the Expression of EMT-Related Proteins

**Figure 6:**
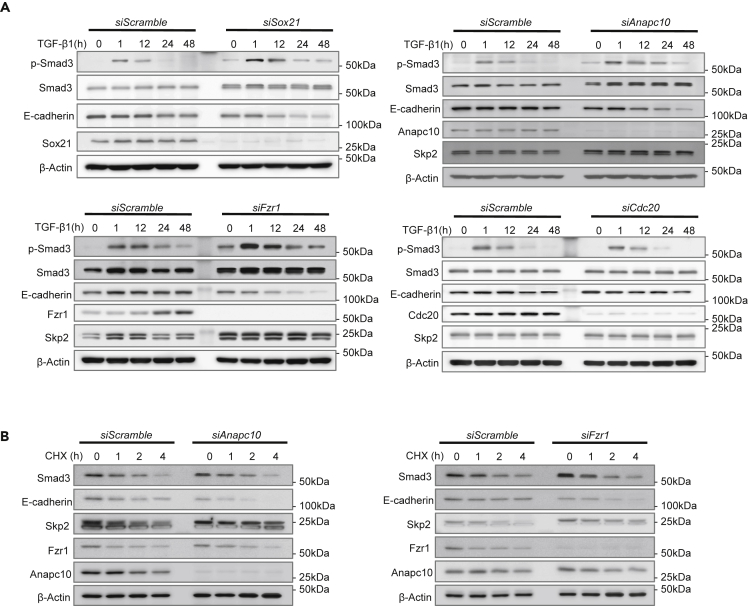
Regulation of EMT in SF2 Cells by Knockdown of Anapc10, Fzr1, and Cdc20 (A) SF2 cells were transfected with *Sox21*, *Anapc10*, *Cdc20*, or *Fzr1* siRNA probes. After 48 h, cells were cultured with TGF-β1 from 0 to 48 h and then processed for immunostaining. (B) siRNA-transfected cells were treated with 20 μg/mL cycloheximide (CHX) to determine the half-life of the indicated proteins. The cells were collected at the indicated time points for western blotting.

Anapc10 is a core component of the APC/C multi-subunit E3 ligase complex and is implicated in substrate recruitment to the complex by functioning as a substrate recognition module together with a co-activator protein, cell division cycle 20 (Cdc20) or CDC20 homolog 1 (Cdh1/Fzr1) (da Fonseca et al., 2011; Kominami et al., 1998). Thus, to investigate the contribution of APC/C activity to the acquired EMT phenotypes elicited by Sox21 deficiency in mice, we conducted an immunoblot analysis to analyze the abundance of the EMT-related proteins Smad3 and E-cadherin in SF2 cells upon knockdown of *Sox21*, *Fzr1*, *Cdc20*, or *Anapc10*. Knockdown of *Sox21*, *Fzr1,* or *Anapc10*, but not *Cdc20*, caused increase in Smad3 protein and induced Smad3 phosphorylation by TGF-β (Figure 6A). Furthermore, we observed an increase in N-cadherin and a reduction in E-cadherin upon *Fzr1* or *Sox21* knockdown in the SF2 cells (Figure S7D).

**Figure 7:**
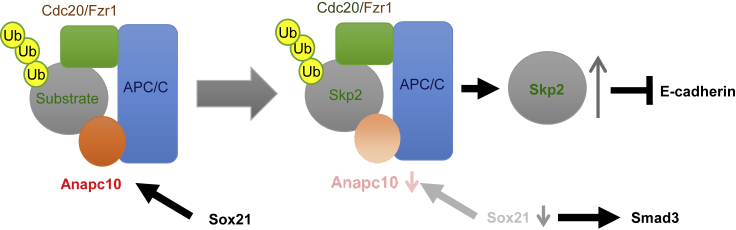
EMT Schematic Diagram of the Relationships among Sox21, Anapc10, Skp2, and E-cadherin Sox21 enhances Smad3, but not Smad 6 and 7. Furthermore, as Sox21 induces Anapc10, loss of Sox21 leads to a reduction in Anapc10. Reduction of the complex containing Anapc10 and Fzr1 induces an increase in Skip2 and indirectly decreases E-cadherin.

In addition, we identified a canonical D-box motif (RxxLxxxxN), which could be targeted by APC/C, in the Smad3 protein, prompting us to test the possibility that APC/C directly regulates Smad3 protein stability through post-translational modification. Hence, we determined the half-lives of Smad3 protein in *Fzr1* or *Anapc10* knockdown and control cells. No significant changes in the half-life of Smad3 protein following *Fzr1* or *Anapc10* knockdown were detected (Figure 6B). This result implies that Smad3 was not subjected to APC/C-mediated ubiquitination and degradation in this circumstance. In comparison, Skp2, a reported APC/C^Fzr1^ substrate (Bashir et al., 2004; Wei et al., 2004), was accumulated and stabilized following the depletion of Fzr1 or Anapc10 in SF2 cells (Figure 6B). Furthermore, consistent with a previous report showing Skp2-mediated E-cadherin degradation (Inuzuka et al., 2012), the protein levels and half-lives of Skp2 and E-cadherin displayed inverse correlations upon *Fzr1* or *Anapc10* knockdown in SF2 cells (Figure 6B). These findings indicate that Fzr1 and Anapc10, both of which play a crucial role in APC/C substrate recognition, regulate EMT by modulating the abundance and activity of EMT-related proteins such as Smad3, Skp2, and E-cadherin (Figure 7).

### Knockdown of *Sox21*, *Fzr1*, or *Anapc10* Promotes TGF-β-Induced E-Cadherin Downregulation

**Figure 8:**
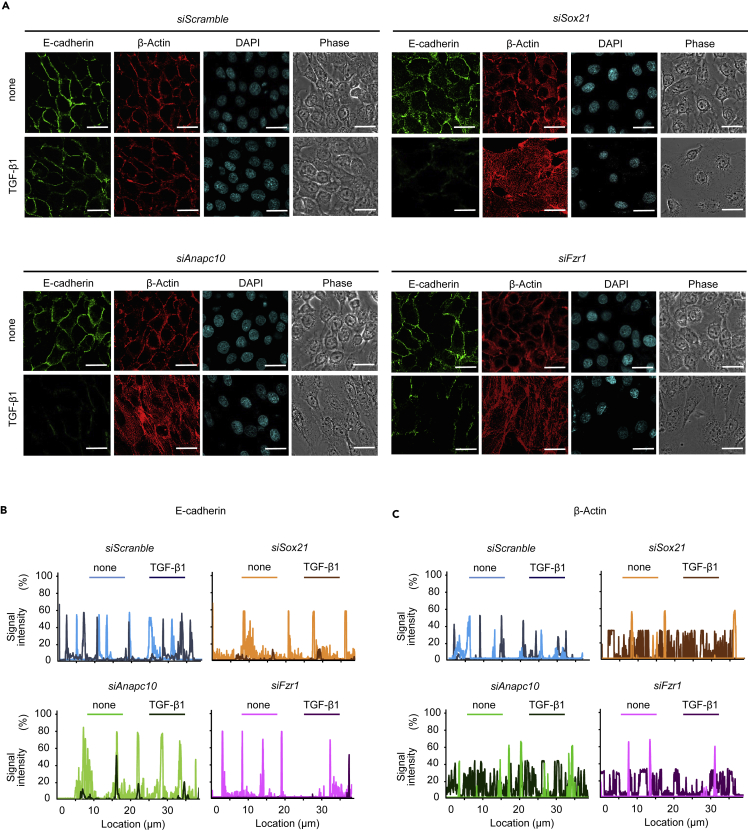
Abolishment of the Dental Epithelium Cytoskeleton by Sox21 and Anapc10 Suppression with TGF-β (A) SF2 cells in which Fzr1, Anapc10, or Sox21 was depleted by siRNA were cultured in the presence or absence of TGF-β1 for 48 h. Cells were stained with anti-E-cadherin antibody (green), anti-β-actin antibody (red), and DAPI (aqua). The protein localization was analyzed by confocal fluorescence microscopy. Scale bar, 10 μm. (B and C) Fluorescence intensity of E-cadherin (B) and β-actin (C) as evaluated with the ImageJ software (NIH).

To further investigate the effects of *Sox21* knockdown in the induction of EMT, we sought to analyze E-cadherin protein levels in *Sox21, Fzr1, Cdc20*, or *Anapc10*-depleted SF2 cells. Both E-cadherin and actin were largely confined to the cell membrane in control cells, as is normally observed in tooth epithelial cells in unstimulated conditions (McCrea et al., 1991) (Figure 8A). Even following TGF-β stimulation, E-cadherin and β-actin abundance on the cell membrane was not significantly altered, indicating that dental epithelial cells are not responsive to TGF-β stimulation under normal conditions (Figure 8). Conversely, knockdown of *Sox21*, *Anapc10*, or *Fzr1*, but not *Cdc20*, resulted in the downregulation of E-cadherin, which was further promoted upon TGF-β stimulation (Figures 8A, 8B, and S7E). Furthermore, we analyzed EMT marker expression in dental epithelial cells after transfection with *Sox21* or *Anapc10* siRNA cultured with TGF-β1 to induce EMT. EMT markers *N-cadherin*, *Vimentin*, *Zeb1*, *Zeb2,* and *Snail1* were not induced by siRNA for *Sox21* or *Anapc10* in NT-4-induced differentiated ameloblasts (Figure S7B), but were induced in undifferentiated immature dental epithelium (Figure S7A). These experiments revealed that immature dental epithelium, but not differentiated ameloblasts, underwent *trans*-differentiation into hair cells and may induce EMT to form hair papilla. Furthermore, upon *Sox21*, *Anapc10*, or *Fzr1* knockdown, β-actin was localized to the cell membranes in the absence of TGF-β1, whereas it was abundantly localized throughout the cells in the presence of TGF-β1 (Figures 8A and 8C). Therefore, these data suggested that Sox21-induced *Anapc10* expression represents a crucial element for suppressing EMT in dental epithelium, and that dysregulation of the inhibitory mechanism may cause an aberrant EMT, leading to abnormal formation of an ameloblast-derived root sheath analog.

## Discussion

Our study revealed a role of Sox21 during ameloblast differentiation. We showed that *Sox21* is expressed in ameloblasts, which differentiate from Sox2-expressing stem cells in dental epithelium (Juuri et al., 2012). Notably, Sox2 and Sox21 have been proposed to have a reciprocal relationship in the nervous system (Argenton et al., 2004; Sandberg et al., 2005). Sox2 expression in the incisor was observed in the cervical loop region and then decreased during dental epithelium differentiation in both wild-type and *Sox21* KO mice (Figure S3). Furthermore, Sox2-positive dental epithelium re-appeared at a later stage in *Sox21* KO incisors (Figure S3 arrowhead), indicating that Sox21 may inhibit Sox2 expression and form the ectopic hair organ. Some cells in the secretory stage of ameloblast start invagination and form a hair organ-like structure. Some pre-ameloblasts may have been retained as ameloblast lineage cells, whereas others switched to hair-forming cells. EdU labeling in the incisor revealed that Sox2-positive cells observed in *Sox21* KO mice did not proliferate and may therefore not generate progeny (Figure S3). Expression of many keratins was increased in the P1 mouse dental epithelium (Figure 3C), which was histologically unchanged (Figures S4A and S4C). Similar results were obtained with the immature ameloblast cell line (Figure 4E). The preparation of genes to make hair may already start from this time. In the incisor, epithelial invasion of a hair-like structure appeared at P4 weeks and hair was formed at P6 weeks (Figures S4D, S4E, and S5A). Lgr5 is expressed in hair germs and the hair bulge that makes up the hair root sheath wall, whereas Sox2 is expressed in the dermal papilla located at the bottom. Immunohistochemistry of Lgr5 and Sox2 in the 6-week-old *Sox21* KO incisor showed a similar expression pattern to that in hair roots (Figures 3A and S6A). The expression of these molecules, which is absent in ameloblasts, indicates that the ameloblasts were transformed into hair-forming cells. Sox2-positive cells are presented in some pre-ameloblasts and stratum intermedium, which may form ectopic hairs in the tooth germ. In the molar, lose polarization was observed in P7-day-old *Sox21* KO mice (Figure S4B). However, we did not observe ectopic hairs in the molar region. The molar started eruption at P2 weeks, and dental epithelial cells were lost after this eruption. From this situation, it is possible that the molars did not have hair structures because they did not have sufficient time to grow. Previously, we showed that the basement membrane component nephronectin and NKX2-3 inhibit Sox2 expression in the dental epithelium (Arai et al., 2017; Han et al., 2018). However, nephronectin was downregulated in differentiated ameloblasts. Both Sox21 and nephronectin may inhibit Sox2 expression in the dental epithelium. Because *Sox21*^+^ and *Sox2*^+^ cell populations are not in close proximity in the incisor epithelium, they do not interact directly. However, our results suggested that Shh activity occurs directly upstream of *Sox21* expression in ameloblasts. By using the predictive algorithm JASPAR, we identified that the main transcription factors predicted to bind upstream of the Sox21-coding sequence were Znf263, Egr1, and Gli2, of which Gli2 is activated by the Shh pathway. It was reported that Hedgehog signaling acts upstream of Sox2 in cancer stem cells (Gopinath et al., 2013), and Sox18 is a target gene of hedgehog signaling in cervical carcinoma (Petrovic et al., 2015). Therefore, we hypothesized that Shh may regulate *Sox21* expression via Gli2 activity. Sox2-expressing cells in tooth epithelium have been shown to be targets of Shh, based on their Gli1 expression (Li et al., 2015). Shh induced *Sox21* expression, and inhibitor of Shh signaling inhibited *Sox21* expression (Figures S1B and S1C). From this result, Shh may be an upstream molecule of Sox21. The deletion of *Shh* shows no hair formation and presence of abnormal follicular structures (St-Jacques et al., 1998). Shh is important for the invagination of the tooth and hair epithelium. The deletion of Shh signaling may inhibit initial tooth and hair formation. Previous reports showed that ectopic hairs were observed in *Med1* and *FAM38h* KO incisors (Wang et al., 2016c; Yoshizaki et al., 2014). Med1 regulates Notch1 and Ca^2+^ channel expression and is involved in ectopic hair formation. However, unaltered expression of Med1, FAM38h, Notch1, and Ca^2+^ channel in *Sox21* KO mice indicated that Sox21, Med1, and FAM38h have independent functions in ectopic hair formation. Furthermore, the hair follicle structure of ectopic hair was unclear in *Med1* KO mice. The hair follicle structure in ectopic hairs of teratomas and dermoid cysts were also unclear (Ahmad et al., 2013; Komiyama et al., 2002; Lewis et al., 2012; Takeda et al., 2003; Tavares et al., 2018). Our results similarly found unclear hair follicle structures. Thus, the hair follicle with ectopic hair may be immature and obscure.

Sox21 deficiency impaired ameloblast function, leading to decreased enamel volume and defective structure of the remaining matrix. The expression of *Amtn*, *Ambn* and *Enam*, which are important for enamel matrix formation and calcification (Meredith et al., 2014), was decreased. Furthermore, we showed that Sox21 directly regulates the expression of Amtn and Klk4, which are involved in ameloblast maturation and enamel formation (Gasse et al., 2012; Kawasaki et al., 2014). Unexpectedly, when *Sox21* expression was inhibited, ameloblasts lost their dental identity and *trans*-differentiated into hair cells. It is possible that, without a clear terminal differentiation signal, the immature dental epithelium retains the capacity to adopt a different cell fate. SF2 cells differentiated into ameloblasts by NT-4. We analyzed the expression of hair cell markers in both differentiated ameloblasts and immature dental epithelium cells, using SF2 cells after transfection with *Sox21* siRNA. In immature dental epithelium, *Sox21* siRNA inhibited the expression of ameloblast markers and induced expression of hair keratins *Krt24* and *Ker32* (Figures 4D and 4E). However, in NT-4-induced differentiated ameloblasts, Sox21 siRNA inhibited ameloblast marker expression and did not induce hair markers (Figures 4F and 4G), indicating that immature dental epithelium, but not differentiated ameloblasts, could differentiate into hair cells. Interestingly, loss of *Sox21* seemed to favor the EMT process, and it is possible that this led to the formation of dermal papilla. The acquisition of migratory properties is a prerequisite for cancer invasion into surrounding tissues. The acquisition of migration requires a dramatic morphologic alteration, termed EMT, such as loss of epithelial characteristics of cell polarity and cell adhesion (Thiery et al., 2009). We speculate that the epithelial characteristic was lost by lack of Sox21, because the dental epithelial arrangement was disrupted and cell polarity was lost after 4 weeks in the *Sox21* KO mice (Figure S4D). Therefore, we analyzed EMT marker expression in dental epithelial cells after transfection with *Sox21* siRNA. EMT markers, *N-cadherin*, *Vimentin*, *Zeb1*, *Zeb2*, and *Snail1*, were not induced by siRNA for *Sox21* in differentiated ameloblasts, but were induced in immature dental epithelium (Figures S7A and S7B). It was reported that various cell adhesion molecules, such as cadherin, contribute to tooth differentiation (Chiba et al., 2019; Saito et al., 2015; Verstraeten et al., 2013; Yamada et al., 2016). These experiments revealed that immature dental epithelium, but not differentiated ameloblasts, *trans*-differentiated into hair cells and may induce EMT to form hair papilla. However, further experiments are needed to demonstrate whether the mesenchymal cells of the dermal papilla actually originated from the immature epithelial ameloblasts via EMT in the *Sox21* KO teeth. It is also possible that Sox2 positivity in the forming dermal papilla mesenchyme was induced by the epithelium after it had adopted hair fate by *Sox21* deletion.

EMT not only is induced by TGF-β signaling but also results from Sox2 activity (Gao et al., 2015; Wang et al., 2016b) and BMP (Dudley et al., 1995; Rothhammer et al., 2005), canonical Wnt (Kemler et al., 2004), and Notch (Xie et al., 2012) signaling. In the tooth, EMT can reportedly occur by the cessation of BMP and Wnt signaling, leading to a switch from crown to root formation (Yang et al., 2013). However, in the present study, the epithelium did not differentiate to mesenchymal structures, but generated the epithelial cell network covering the root (epithelial cell rests of Malassez). In turn, TGF-β activity is important for tooth formation and cell differentiation (Vaahtokari et al., 1991). Specifically, TGF signaling in ameloblasts (Khan et al., 2013; Zhu et al., 2000) is important for enamel formation as its inhibition can lead to amelogenesis imperfecta (Huckert et al., 2015; Poche et al., 2012; Zhao et al., 2011). Thus, TGF activity does not normally induce EMT in the dental epithelium. This conclusion is supported by our result demonstrating the maintenance of E-cadherin expression in SF2 cells upon the addition of TGF-β1 (Figure 8A). However, TGF-β activity was enhanced by decreases in Sox21 and Anapc10, leading to EMT induction *in vitro*. Furthermore, signs of EMT, i.e., the expression of Vimentin, and N-cadherin, were observed *in vivo* upon Sox21 deficiency. Thus, our results suggest that the maintenance of ameloblast identity and their proper differentiation depend on *Sox21* expression. However, EMT did not occur in NT-4-induced differentiated ameloblasts *in vitro* (Figures S7A and S7B), although it may have occurred in the immature dental epithelium of *Sox21* KO mice.

Our findings demonstrate that, among the Sox21 direct targets, the expression of *Anapc10* is crucial in the ameloblast differentiation process. Anapc10 is one of the core subunits of APC/C, the multi-subunit RING finger E3 ubiquitin ligase that regulates exit from mitosis through the degradation of various mitotic regulators such as cyclin B and Securin (Carroll and Morgan, 2002; Kominami et al., 1998; Passmore et al., 2003). Here, we showed that Sox21 and Anapc10 negatively regulate Smad3 activity. Upon being relieved from this regulation, the increased Smad3 activity led to a potentiated TGF response toward EMT in the dental epithelium. Anapc10 has been reported to form a substrate recognition module together with Fzr1 or Cdc20 to recognize the D-box motif present in substrates for ubiquitination and subsequent degradation (Kominami et al., 1998). Our results suggest that Anapc10 and Fzr1 positively regulate E-cadherin and negatively regulate Smad3, N-cadherin, and Skp2 at the protein level. Furthermore, Anapc10 directly binds to Sox2 with ubiquitin-conjugating enzyme Ube2c and regulates Sox2 stability (Wang et al., 2016a). These observations suggest that Anapc10, together with Fzr1, prevents EMT in the dental epithelium and may regulate Sox2 expression. Our data further indicate that although Smad3 contains a consensus D-box motif sequence, it is unlikely that APC/C^Fzr1^ directly ubiquitinates Smad3 for degradation (Figures 5C, 5D, 6, S7C, and S7D). Instead, the previous observation (Nourry et al., 2004) that Anapc10 directly binds to Smad3 to trigger the ubiquitination of NEDD9/HEF1, a critical positive regulator of EMT, suggests that the EMT elicited by *Sox21* KO in the dental epithelium may stem from a stabilization of NEDD9/HEF1 partly through decreased *Anapc10* expression and impaired APC/C activity (Beck et al., 2014; Morimoto et al., 2014).

In addition, we present evidence that *Anapc10* knockdown led to Skp2 stabilization and the concomitant destabilization of E-cadherin in SF2 cells (Figure 6). As previous studies have demonstrated that APC/C^Fzr1^ degrades Skp2 (Bashir et al., 2004; Wei et al., 2004) and that Skp2 directs E-cadherin degradation (Inuzuka et al., 2012), our data support the hypothesis that the Anapc10/Fzr1/Skp2/E-cadherin signaling pathway contributes to the EMT observed in *Sox21* KO dental epithelium. During tooth and hair follicle development, ectodermal cells form a bud structure by changing their polarity and cell-cell contacts via E-cadherin. The transcription factor Lef1 downregulates E-cadherin to induce epithelial bud invagination. Moreover, forced elevation of E-cadherin expression blocks invagination and hair follicle production (Jamora et al., 2003). Overexpression of Lef1 under the control of the K14 promoter induced hair formation in the incisor region similar to our observation in Sox21-deficient mice (Zhou et al., 1995). These results suggest that downregulation of E-cadherin may be critical for tooth hair formation.

Taken together, our data suggest the existence of ameloblast plasticity that is maintained under the control of Sox21. Sox21 inhibited dental epithelial EMT and downregulation of E-cadherin via Anapc10. This information is useful for applications in the regeneration of organs including teeth and hair.

### Limitations of the Study

This study reveals a role for Sox21 in ameloblasts. Inhibition of Sox21 switched the cell fate of dental epithelium to hair cells. Furthermore, the dental epithelium undergoes EMT and changes into mesenchymal cells. However, this may be limited to the incisors in rodents that continue to erupt. On the other hand, it may contribute to the elucidation of the differentiation of other ectodermal organs and may lead to the progress of the function of Sox21 expressed in the neural crest because tooth cells are derived from the neural crest.

### Resource Availability

#### Lead Contact

Further information and requests for resources and reagents should be directed to and will be fulfilled by the Lead Contact, Kan Saito (kanta@dent.tohoku.ac.jp).

#### Materials Availability

This study did not generate new unique reagents.

#### Data and Code Availability

The data that support the findings of this study are available from the Lead Contact on reasonable request. Microarray data have been deposited in NCBI's Gene Expression Omnibus (GEO) and are accessible through GEO series accession number GSE99359 (https://www.ncbi.nlm.nih.gov/geo/query/acc.cgi?acc=GSE99359), GSE99360 (https://www.ncbi.nlm.nih.gov/geo/query/acc.cgi?acc=GSE99360).

## Methods

All methods can be found in the accompanying Transparent Methods supplemental file.

## Supplemental Information

Document S1. Transparent Methods, Figures S1–S7, and Tables S1 and S2
