# Supplementary material for: Sox21 Regulates Anapc10 Expression and Determines the Fate of Ectodermal Organ
Source: iScience. 2020 Jun 30;23(7):101329. doi: 10.1016/j.isci.2020.101329 (PMC7363706; doi:10.1016/j.isci.2020.101329)
Supplement: Document S1. Transparent Methods, Figures S1–S7, and Tables S1 and S2 [file mmc1.pdf]

iScience, Volume *23*

Supplemental Information

Sox21 Regulates Anapc10 Expression and Determines the Fate of Ectodermal Organ

Kan Saito, Frederic Michon, Aya Yamada, Hiroyuki Inuzuka, Satoko Yamaguchi, Emiko Fukumoto, Keigo Yoshizaki, Takashi Nakamura, Makiko Arakaki, Yuta Chiba, Masaki Ishikawa, Hideyuki Okano, Irma Thesleff, and Satoshi Fukumoto

Figure S1

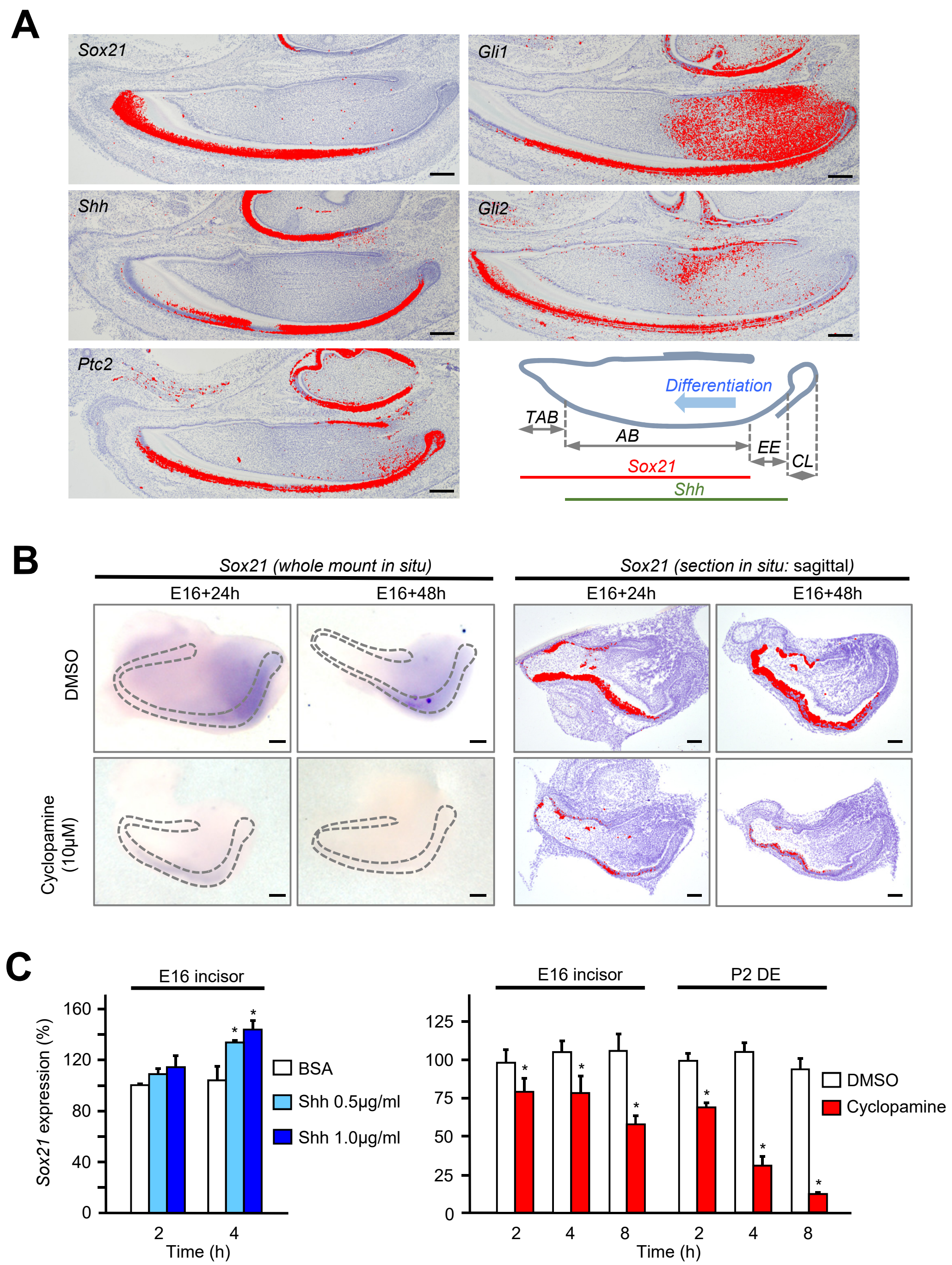
A
Sox21
Gli1
Shh
Gli2
Ptc2
Differentiation
TAB
AB
EE
CL
Sox21
Shh
B
Sox21 (whole mount in situ)
Sox21 (section in situ: sagittal)
E16+24h
E16+48h
E16+24h
E16+48h
DMSO
Cyclopamine (10μM)
C
E16 incisor
Sox21 expression (%)
160
120
80
40
0
BSA
Shh 0.5μg/ml
Shh 1.0μg/ml
2
4
Time (h)
E16 incisor
P2 DE
125
100
75
50
25
0
DMSO
Cyclopamine
2
4
8
2
4
8
Time (h)

Figure S1. Expression and effect of Shh-related genes in the tooth. Related to Figure 1.

(A) Radioactive *in situ* hybridization on sagittal sections of the lower incisor from 6-week-old mice. ^{35}S-labeled RNA probes were used to detect the expression of *Sox21*, *Shh*, *Ptc2*, *Gli1*, and *Gli2*. Ptc2 of the receptor and Gli of the intracellular signal were used for analysis of Shh signaling. Scale bar, 200 μm. CL, cervical loop. EE, enamel epithelium. AB, ameloblasts. TAB, terminal ameloblasts.

(B) Incisors were dissected from E16-stage mice under a stereomicroscope. Tissues were cultured using a Trowell organ culture system for 24 or 48 h with the Shh inhibitor cyclopamine (10 μM). Whole-mount *in situ* hybridization using digoxigenin-labeled probes was performed. Radioactive *in situ* hybridization on sagittal sections of the lower incisor was carried out in accordance with standard protocols. Scale bar, 200 μm.

(C) E16 tooth germs or P2 dental epithelium (DE) were cultured with recombinant Shh or cyclopamine. *Sox21* expression was quantified by qPCR and was normalized to that of *Gapdh* expression. The expression in the control sample was set to 100 and expression in the experimental samples was determined as the fold change relative to the control using the ΔΔCT method. Error bars represent mean ± SEM of five technical replicates. *$p < 0.05$, Student's *t*-test.

Figure S2

A

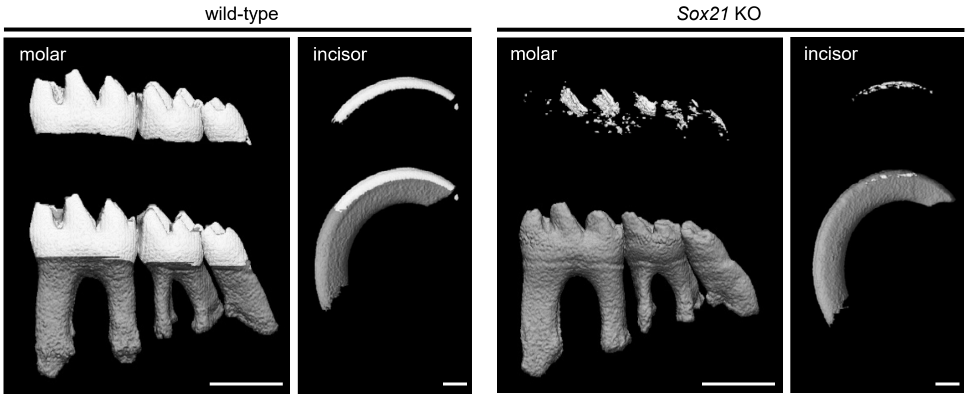

B

		Volume (mm³)		Fold change
		wild-type	*Sox21* KO	(*Sox21* KO / wild-type)
incisor	Enamel	0.1617349	0.0141242	0.0873293
	Dentin	1.8942210	2.1707440	1.1459824
molar	Enamel	0.3113039	0.0187767	0.0603163
	Dentin	1.2306690	1.2484150	1.0144198

Figure S2. Micro-computed tomography (micro-CT). Related to Figure 2.

Molars and incisors of 6-week-old wild-type and *Sox21* KO mice were analyzed by micro-CT. Mineralization volumes (mm^3) of enamel and dentin were calculated. The tooth enamel and dentinal quantity were determined by the density of mineralization.

(A) Calcium scores of incisors and molars scanned by micro-CT. The lower figure is the whole tooth and the upper figure is the enamel. Scale bar, 1mm.

(B) Tooth enamel and the dentin-cementum were divided on the basis of the difference in calcification density, and the volumes were measured. Fold changes were calculated from the differences in density between wild-type (WT) and *Sox21* KO (KO) mice samples.

Figure S3

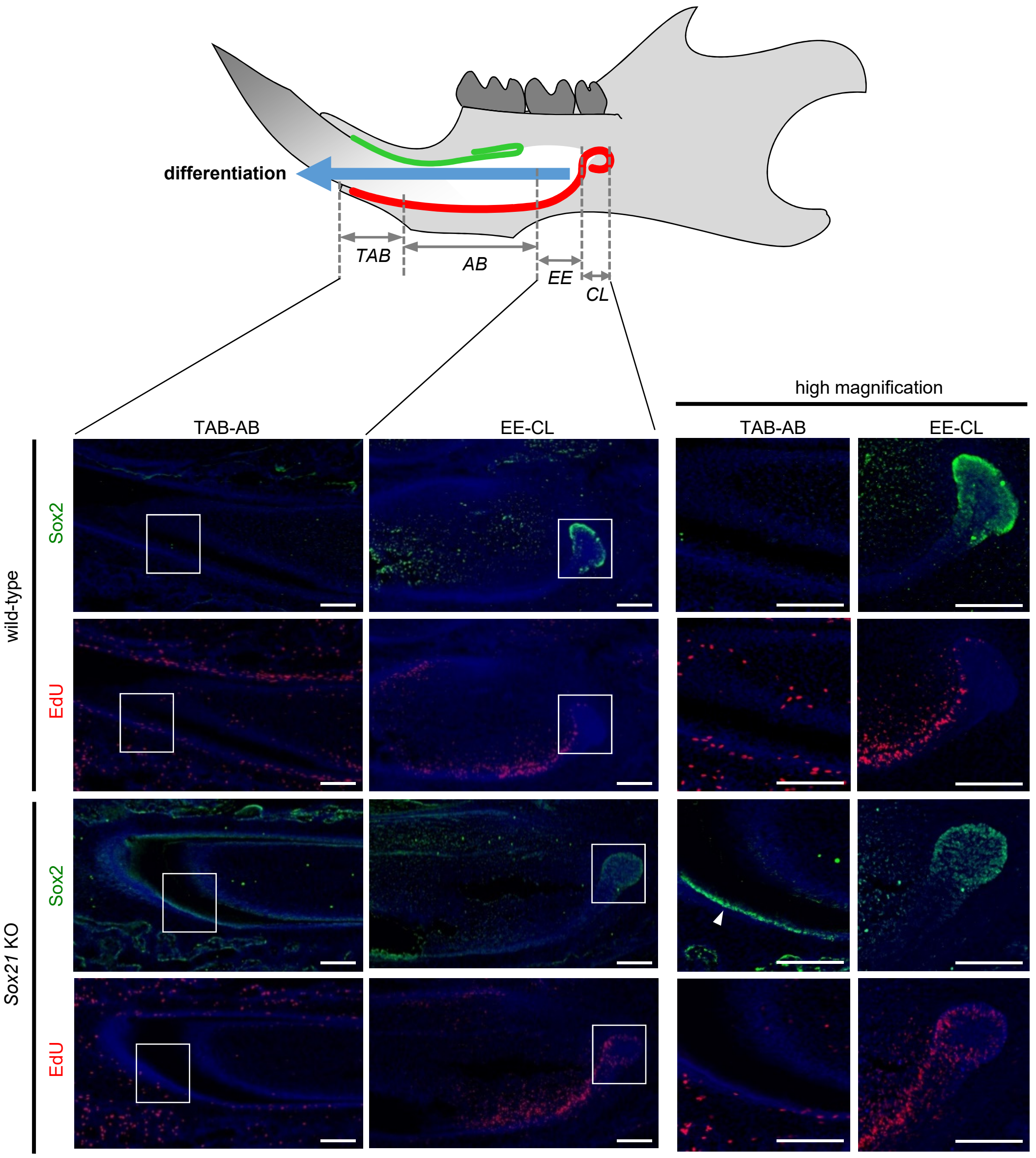

differentiation
TAB
AB
EE
CL
high magnification
TAB-AB
EE-CL
TAB-AB
EE-CL
wild-type
Sox2
EdU
Sox21 KO
Sox2
EdU

Figure S3. Sox2 expression and cell proliferation assay in *Sox21* KO mouse incisors. Related to Figure 3.

Schematic of a sagittal section through the mouse incisor. The cervical loop (CL) exists in the posterior region of the incisor. The enamel epithelium (EE) differentiates sequentially into ameloblasts (AB) and terminal ameloblasts (TAB), while cells migrate anteriorly from the labial CL. The red signal is EdU; green signal, Sox2 immunostaining. The right panel shows these results at high magnification. Scale bar, 200 μm.

Figure S4

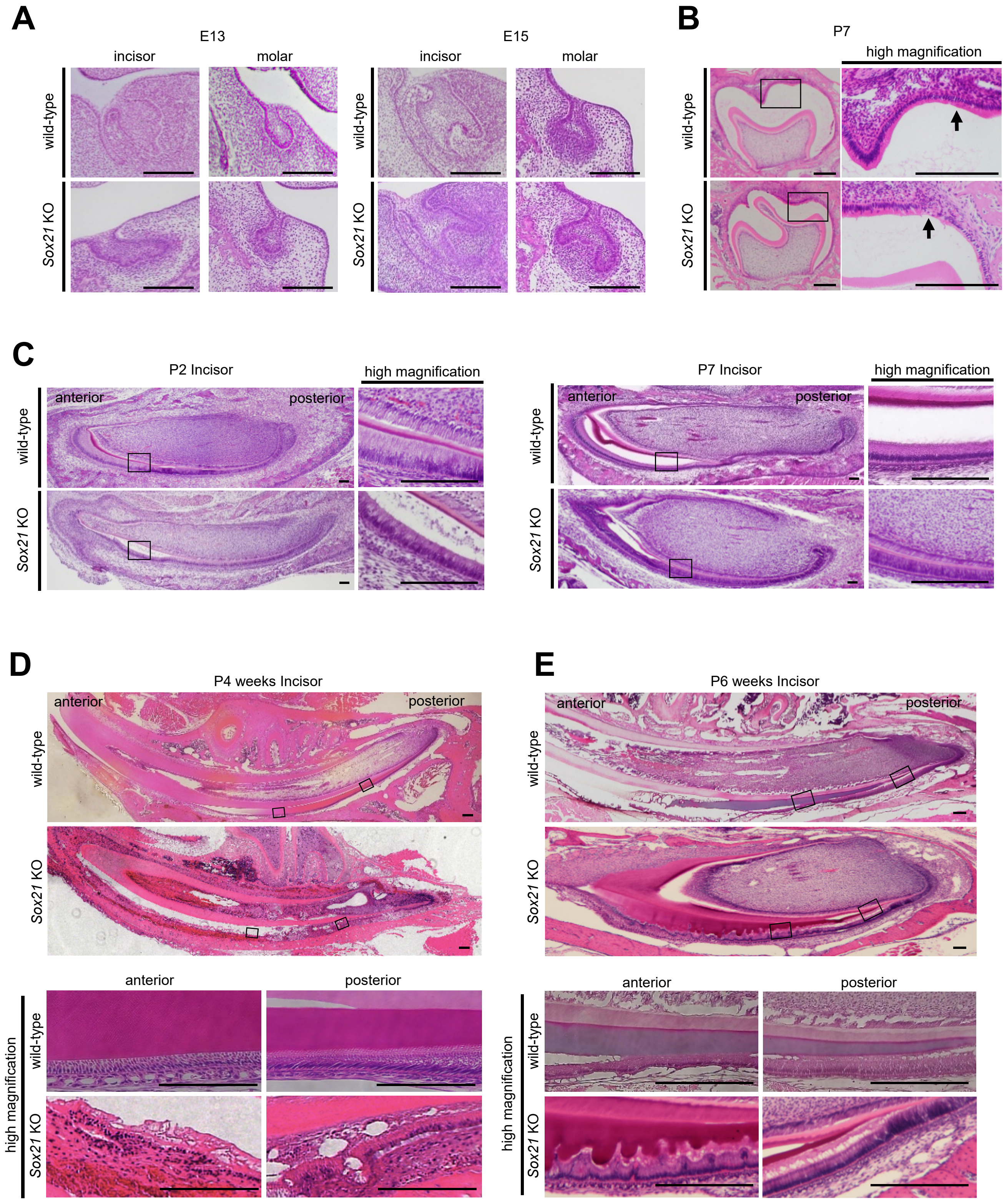
A
E13
incisor
molar
E15
incisor
molar
wild-type
Sox21 KO
B
P7
high magnification
wild-type
Sox21 KO
C
P2 Incisor
high magnification
anterior
posterior
P7 Incisor
high magnification
anterior
posterior
wild-type
Sox21 KO
D
P4 weeks Incisor
anterior
posterior
wild-type
Sox21 KO
anterior
posterior
high magnification
wild-type
Sox21 KO
E
P6 weeks Incisor
anterior
posterior
wild-type
Sox21 KO
anterior
posterior
high magnification
wild-type
Sox21 KO

Figure S4. Histological analysis of the wild-type and *Sox21* KO mice. Related to Figure 3.

(A) Incisors and molars in the embryonic stage of wild-type and *Sox21* KO mice were stained with hematoxylin and eosin (H-E). The left panel shows a sagittal section of the incisor; right panel, coronal section of the molar.

(B) The molar of 7-day-old wild-type and *Sox21* KO mice were stained with H-E. The right panel show high magnification of ameloblasts in a molar.

(C) The incisors of 2 and 7 days post-natal wild-type and *Sox21* KO mice were sectioned in a sagittal plane. The right panel shows these results at high magnification.

(D, E) The incisors of 4- and 6-week-old mice were sectioned in a sagittal plane. The lower panel shows these results at high magnification. Scale bar, 200 μm.

Figure S5

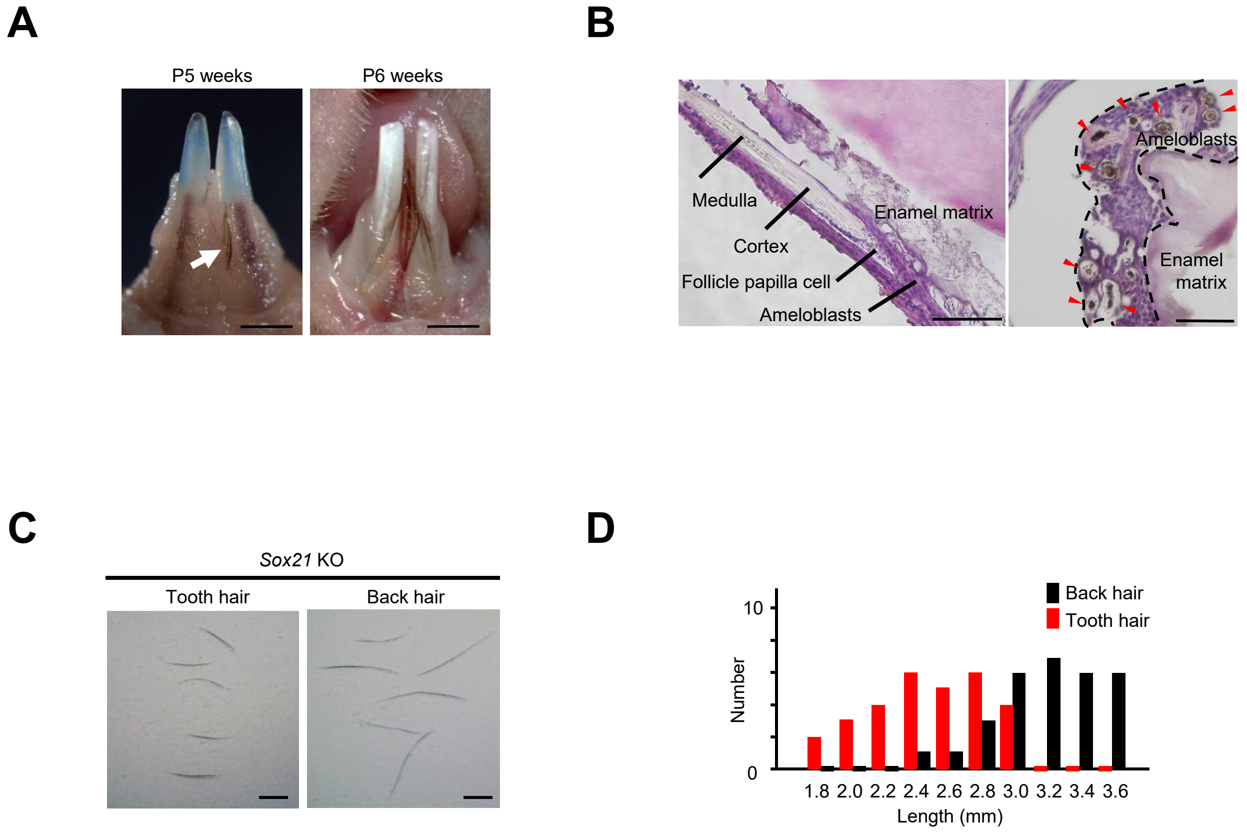
A
P5 weeks
P6 weeks
B
Medulla
Cortex
Follicle papilla cell
Ameloblasts
Enamel matrix
Ameloblasts
Enamel
matrix
C
Sox21 KO
Tooth hair
Back hair
D
Back hair
Tooth hair
10
0
Number
1.8 2.0 2.2 2.4 2.6 2.8 3.0 3.2 3.4 3.6
Length (mm)

Figure S5. Anatomical observations of tooth hairs in *Sox21* KO mice. Related to Figure 3.

(A) For observation of tooth hairs, 5- and 6-week-old *Sox21* KO mouse lower incisor gingiva were assessed with a stereomicroscope. Arrow indicates hair in the gingiva. Scale bar, 1 mm.

(B) Histological analysis of the 6-week-old *Sox21* KO mouse lower incisor. Left panel shows sagittal section, right panel shows frontal section. Red arrowheads indicate the hair root sheath structure. Scale bar, 100 μm.

(C, D) The tooth and back hairs of 6-week-old *Sox21* KO mice were examined by stereomicroscopy. Stereomicroscopy was used to investigate hair morphology (C). Scale bar, 1 mm. Thirty hairs on each tooth and back were measured (D).

Figure S6

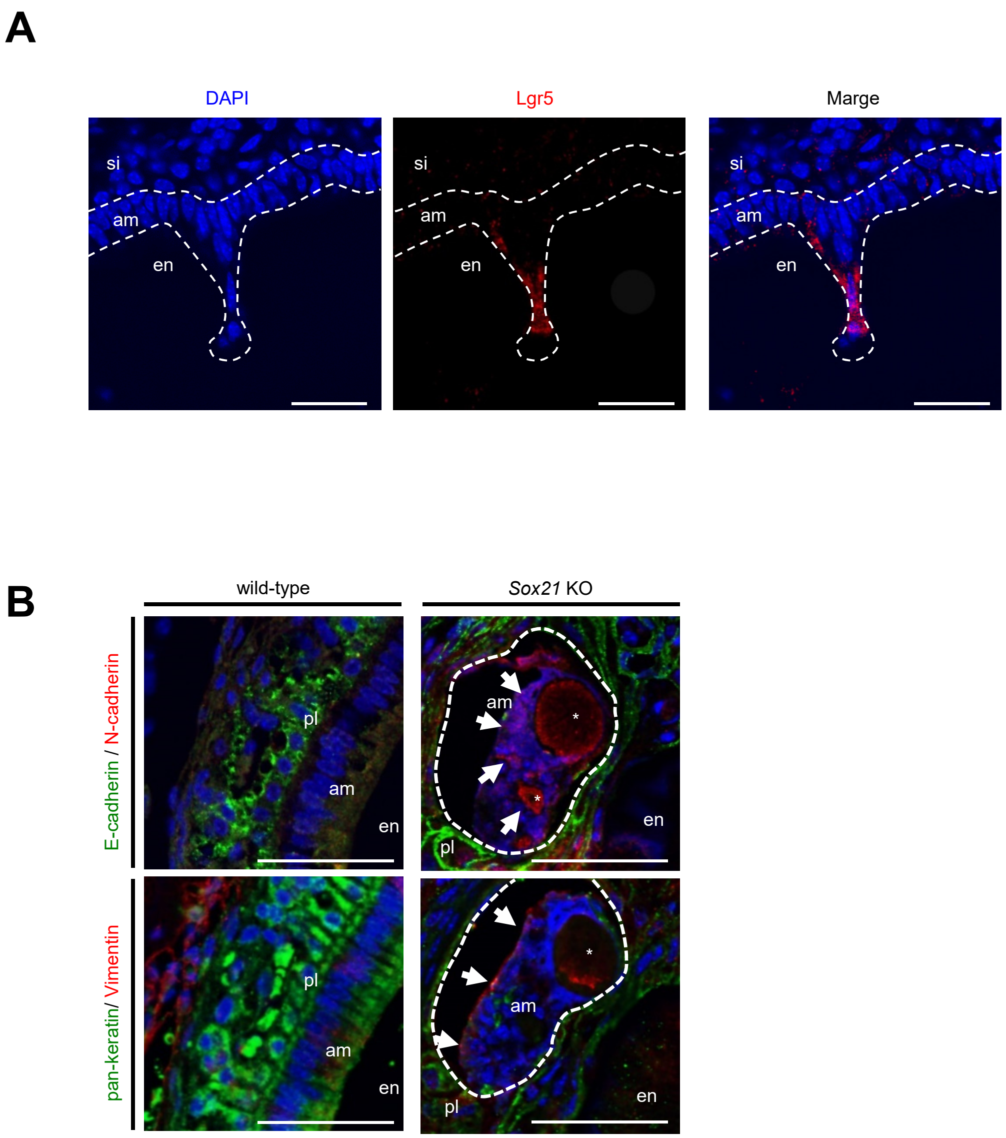
A
DAPI
Lgr5
Marge
si
am
en
B
wild-type
Sox21 KO
E-cadherin / N-cadherin
pan-keratin/ Vimentin
pl
am
en
*

Figure S6. Immunohistochemistry of hair root sheath structures derived from odontogenic epithelium. Related to Figure 3.

(A) The ameloblasts of 6-week-old *Sox21* KO mouse incisors were immunostained using anti-Lgr5 antibody with DAPI. si, stratum intermedium; am, ameloblasts; en, enamel. Scale bar, 100 μm.

(B) Double immunostaining of incisal frontal section from a 6-week-old mouse, counterstained with DAPI. Upper panels show sections stained using anti-E-cadherin and anti-N-cadherin antibodies; lower panels, anti-pan-keratin and anti-Vimentin. Scale bar, 100 μm. am, ameloblast; en, enamel. pl, papillary layer; arrow, mesenchyme marker positive cells; *root sheath.

Figure S7

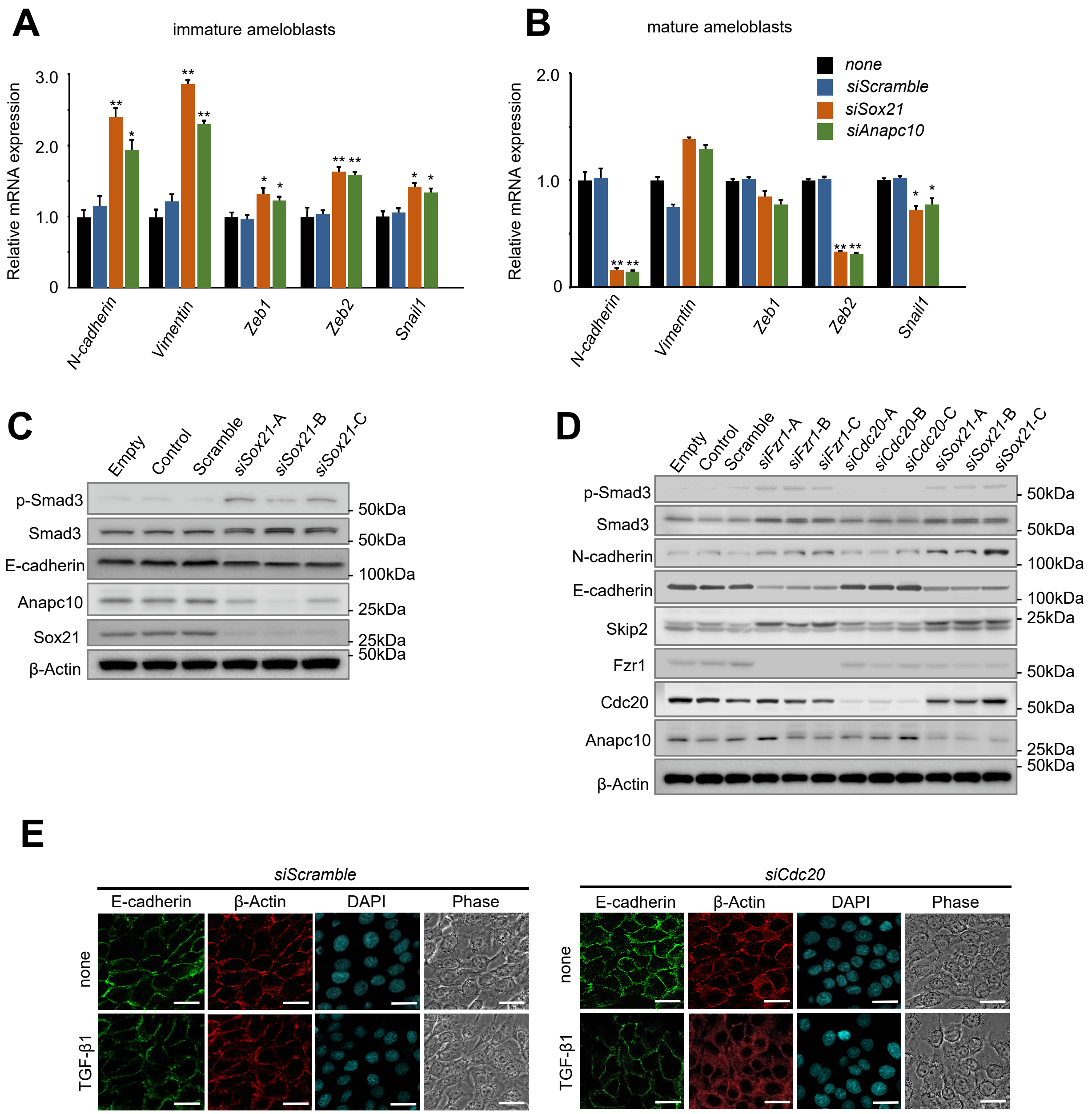

A
immature ameloblasts
Relative mRNA expression
3.0
2.0
1.0
0
N-cadherin
Vimentin
Zeb1
Zeb2
Snail1
B
mature ameloblasts
none
siScramble
siSox21
siAnapc10
Relative mRNA expression
2.0
1.0
0
N-cadherin
Vimentin
Zeb1
Zeb2
Snail1
C
Empty
Control
Scramble
siSox21-A
siSox21-B
siSox21-C
p-Smad3
Smad3
E-cadherin
Anapc10
Sox21
β-Actin
50kDa
50kDa
100kDa
25kDa
25kDa
50kDa
D
Empty
Control
Scramble
siFzr1-A
siFzr1-B
siFzr1-C
siCdc20-A
siCdc20-B
siCdc20-C
siSox21-A
siSox21-B
siSox21-C
p-Smad3
Smad3
N-cadherin
E-cadherin
Skip2
Fzr1
Cdc20
Anapc10
β-Actin
50kDa
50kDa
100kDa
100kDa
25kDa
50kDa
50kDa
25kDa
50kDa
E
siScramble
siCdc20
E-cadherin
β-Actin
DAPI
Phase
none
TGF-β1

Figure S7. Induction of EMT by inhibition of Sox21 related genes. Related to Figure 4.

(A, B) *Sox21* or *Anapc10* expression in SF2 cells was repressed using siRNA. Scramble siRNA was used as a negative control. After 48 h, the expression of *N-cadherin*, *Vimentin*, *Zeb1*, *Zeb2*, and *Snail1* was evaluated by qPCR. SF2 cells were used as rat immature ameloblasts (A). SF2 cells were cultured with NT-4 for differentiation to mature ameloblasts (B). Error bars represent mean ± SEM of five technical replicates. Student's *t*-test (*$p < 0.05$, **$p < 0.005$).

(C, D) SF2 cells were cultured with the indicated siRNAs for 48 h, harvested in cell lysate buffer with a proteinase inhibitor mixture, and subjected to western blotting. SF2 cells transfected with scramble and three different types (-A, -B, -C) of *Sox21* siRNA (C). SF2 cells were cultured with *Fzr1*, *Cdc20*, and *Sox21* siRNAs (three types for each) (D).

(E) SF2 cells were transfected with scramble or *Cdc20* siRNA probes. Transfected cells were cultured with 1 ng/mL TGF-β1 for 48 h. Fixed cells were incubated with anti-E-cadherin (green) and anti-β-Actin (red) primary antibodies. Expression was detected using Alexa 488- or Alexa 594-conjugated secondary antibodies. Cells were counterstained with DAPI (aqua). Fluorescence microscopy was used for image analysis. Images were prepared using a BZ analyzer.

Table S1

			P1 *Sox21*KO and WT tooth germ			P1 WT tooth germ and skin		
			wild-type	*Sox21* KO	*Sox21*KO /WT	Tooth	Skin	Skin / Tooth
	Gene	ID	normalized	normalized	fold change	normalized	normalized	fold change
**Down regulation**	*Wnt16*	NM_053116	0.545214	0.110121	**0.2019776**	0.07893681	1.01384586	**12.843766**
	Slc24a4	NM_172152	1.485913	0.307624	**0.2070268**	0.39510026	0.35461334	**0.8975275**
	Nfasc	NM_182716	3.5188	0.899331	**0.2555788**	0.77622677	0.1611484	**0.2076048**
	Amtn	NM_027793	13.05109	3.526093	**0.2701761**	1.9298519	0.0180962	**0.009377**
	Gdf5	NM_008109	7.573416	2.282076	**0.3013271**	1.99269568	0.61521532	**0.3087352**
	Gal	NM_010253	4.820243	1.654346	**0.343208**	6.11920868	2.15714656	**0.3525205**
	Lama3	NM_010680	22.29153	8.233238	**0.3693437**	18.1096405	5.62769133	**0.3107567**
	Wnt8b	NM_011720	0.72575	0.345303	**0.4757878**	0.25213238	0.07998837	**0.3172475**
	Lamb3	NM_008484	70.09956	33.37616	**0.4761251**	46.3924779	16.993905	**0.3663073**
	Klk4	NM_019928	46.50384	22.53712	**0.4846291**	10.476001	0.17508592	**0.016713**
**Up regulation**	*Krt6a*	NM_008476	4.328279	10.50092	**2.426119**	5.04476617	3.98179156	**0.7892916**
	Sox2	NM_008476	0.084384	0.204748	**2.4263943**	0.06794838	0.10708674	**1.5760013**
	Serpinb2	NM_011111	0.09823	0.241208	**2.4555493**	0.06468927	2.66754757	**41.236323**
	Eppk1	BC026387	2.439613	5.99995	**2.4593853**	5.0134961	43.1576469	**8.6082937**
	Ifi202b	NM_011940	0.20634	0.509715	**2.4702613**	2.38397697	0.61965206	**0.2599237**
	Aqp3	NM_016689	4.686664	11.58602	**2.4721252**	2.24232905	43.2737428	**19.298569**
	Calcr	NM_007588	0.477696	1.230294	**2.5754752**	0.01744918	0.01597048	**0.9152569**
	Gjb3	NM_001160012	0.463193	1.207677	**2.6072894**	0.93076087	50.1520337	**53.882834**
	Gjb2	NM_008125	0.434406	1.133406	**2.6090954**	0.68838086	25.6058581	**37.197225**
	Dsg3	NM_030596	0.361908	0.948636	**2.621204**	0.37224052	2.7124189	**7.2867374**
	Krt6b	NM_010669	4.638635	12.17714	**2.625157**	2.38215898	7.8478616	**3.2944323**
	Scel	NM_022886	0.341166	0.926424	**2.7154678**	0.84052962	10.2047208	**12.140822**
	Krt1	NM_008473	0.73345	2.006899	**2.7362462**	0.56873695	563.543826	**990.86902**
	Meis2	NM_001159569	0.540254	1.563286	**2.8936115**	0.32500126	0.9318031	**2.8670754**
	Krt23	NM_033373	1.269915	3.877613	**3.053442**	1.16792106	59.0901477	**50.5943**
	Klk13	NM_001039042	0.150493	0.469854	**3.1220977**	0.13142733	4.63268086	**35.248992**
	Krt15	NM_008469	12.11519	39.41645	**3.2534733**	9.01582555	215.751428	**23.930302**
	Calm4	NM_020036	1.18064	4.17527	**3.5364453**	1.32356009	677.933992	**512.20492**
	Lgr5	NM_010195	0.147347	0.52749	**3.579916**	0.24952161	3.94997083	**15.830175**
	Krt10	NM_010660	0.409956	1.470797	**3.5876899**	2.55683595	871.239769	**340.74919**
	Krt13	NM_010662	3.106192	11.88748	**3.8270265**	11.6189001	60.2434714	**5.1849548**
	Spink5	NM_001081180	0.677767	2.765247	**4.0799382**	0.67279899	29.5925682	**43.98426**
	Krt32	NM_001159374	0.113465	0.597378	**5.2648459**	0.1467483	0.75555202	**5.148625**

Table S1. Microarray analysis of ameloblasts in the *Sox21* KO mice Related to Figure 3.

Comparing wild-type (WT) and *Sox21* KO (KO), the molecules with decreased expression are shown in blue, and the molecules with increased expression are shown in red. Comparing skin and tooth of wild-type mice, the Down-regulation molecules are shown in blue, and the Up-regulation molecules are shown in red.

Table S2

Primers for qPCR

Gene	Forward	Reverse
Gapdh	TCCTGCACCACCAACTGCTTA	AGTGATGGCATGGACTGTGGT
Sox21	GGGGCCCGGTTTGTATGT	CAACTGCTGGCGCTAACAAC
Slc24a4	TGATCACCAACAAGTTTGGGC	GCTGTGGGTCTTCAGGGTTC
GDF5	AAAGGGCAAGATGACCGAGG	TAAGATCCGCAGTTCAGCCC
Amtn	CCTTATCCACCCCTTGTTCCC	TCTGTGACASCAGGAGTTGTG
Lamb3	GGAGTCAGAGCTGCTTCGAG	GTAACTGCGGATCTGCTCCA
Klk4	AGCAGCCGGATCATACAAGG	TCTTCTGAGAACAGTGCCGC
Krt24	GTAAGGGGTGGATTCGGAGC	GAGAAGGCCCCCATCATAGC
Krt6	GGAAATTGCCACCTACAGGA	GGTGGACTGCACCACAGAG
Sox2	AATCACAACAATCGCGGCGG	CTGGCGGAGAATAGTTGGGG
Klk13	CAGTGCGCCAACATTGAACT	GCCGTAGAGTTTGCCATTGC
Krt23	AGGATGGCAGTGGATGACTTC	CCTCCTGTTCCAGGTCTGT
Krt1	GCAAACTCAAATCAGCGAAACC	TTGCTCCTCTGGCAAATGCT
Lgr5	GCGTCTTCACCTCCTACCTG	GCTCCCTTGGGAATGTGTGT
Krt32	GTGAGGCAGCTGGAGAAAGA	TCAGGACACATGGTCAGCAC
Krt15	GGCCAGGTCAATGTGGAAATG	TAGACGCCACCTCCTTGTTC
Krt13	CTGGCATTGATCTGACCCGT	CCCTCCGATTCTTCTCTGCC
Krt10	GTGCAGCTCTCCCAGATTCA	TGTTGGTACTCGGCGTTCTG
Kradap	TCCTGAACTGGCACGTCAT	CGGCACTTCTCAGTCCTTTC
Anapc10	TCAGGCTGTYTGGTCACTCT	AGCCATCRGATTGCCAGTAA
N-cadherin	ACAAAGGCAGAAGAGAGACTGG	ATGAAGATGCCCGTTGGAGG
Vimentin	AGGCCGAGGAATGGTACAAG	GACTGCACCTGTCTCCGGTA
Zeb1	CAGGAGGAGCCCCAAGTAGAA	GTTGGCACTTGGTGGGACTAC
Zeb2	GTTGTGATCCTCCTCTCAGGC	TAATGACAGGTCCAACGGCT
Snail1	TCACCTTCCAGCAGCCCTAC	TTGCCACTGTCCTCATCGGA

Table S2. Primer sequences Related to Figure 3.

These primers were used with SYBR Select Master Mix and Step One Plus Real-Time PCR system.

Transparent Methods

KEY RESOURCES TABLE

REAGENT or RESOURCE	SOURCE	IDENTIFIER
Antibodies		
Anti-Sox2	Abcam	Cat# Ab137385
Anti-Ameloblastin	Santa Cruz	Cat# sc-50534, 33100
Anti-Pan-keratin	ThermoFischer	Cat# 18-0059
Anti-Vimentin	GeneTex	Cat# GTX85471
Anti-E-cadherin	R&D systems	Cat# AF748
Anti-N-cadherin	GeneTex	Cat# GTX112734
Anti-β-Actin	Abcam	Cat# mAbcam8226
Anti-Alexa488	ThermoFischer	Cat# A11055/A21206
Anti-Alexa594	ThermoFischer	Cat# A11058/A21207
Anti-Smad3	Cell Signaling Technology	Cat# 9513
Anti-phospho Smad3	Cell Signaling Technology	Cat# 9520
Anti-Smad6	Sigma-Aldrich	Cat# WH0004091M7
Anti-Smad7	Sigma-Aldrich	Cat# SAB2108469
Anti-Sox21	LifeSpan BioSciences	Cat# LS-C107486
Anti-Fzr1	Santa Cruz	Cat# sc56312
Anti-CDC20	Santa Cruz	Cat# sc13162
Anti-Anapc10	Atlas Antibodies	Cat# HPA044547
Anti-Skp2	Santa Cruz	Cat# F1714
Anti-DDK	Origene	Cat# TA50011
Chemicals, Peptides, and Recombinant Proteins		
Cyclopamine	Merck Millipore	Cat# 239803
Sonic hedge hog	R&D Systems	Cat# 464-SH
TGF-β1	R&D systems	Cat# 240-B
NT-4	Alomone Labs	Cat# N-270
cycloheximide	Sigma-Aldrich	Cat# C1988
Superscript Vilo MasterMix	ThermoFischer	Cat# 11755050
SYBR® Select Master Mix	Applied Biosystems	Cat# 4309155
ViaFect™ Transfection Reagent	Promega	Cat# E4981
Critical Commercial Assays		
miRNeasy mini kit	QIAGEN	Cat# 217004
ChIP-IT Express kit	Active Motif	Cat# 53008
ECL Prime kit	GE Healthcare	Cat# RPN2232
Deposited Data		
RNA-seq data of Sox21KO mouse	This paper	GSE99359
RNA-seq data of skin and tooth	This paper	GSE99360
Experimental Models: Cell Lines		
SF2 cells	Arakaki et al., 2012	N/A
mDP cells	Arakaki et al., 2012	N/A

Experimental Models: Organisms/Strains		
C57BL/6J mice	The Jackson Laboratory	JAX: 000664
K14-Follistatin mice	Wankell et al., 2001	N/A
follistatin knockout mice	Matzuk et al., 1995	N/A
Sox21 knockout mice	Kiso et al., 2009	N/A
Recombinant DNA		
pCRII-TOPO	ThermoFischer	Cat# K460001
pEF6/V5-His TOPO	ThermoFischer	Cat# K961020
pCMV6-Entry	Origene	Cat# PS100001
Sequence-Based Reagents		
Primers for Sox21 probe; Forward GATGTATAGGTGTCAGGCA	This paper	N/A
Primers for Sox21 probe; Reverse GGTCATTCACTGGTCAAG	This paper	N/A
Primers for qRT-PCR, see Table S2	This paper	N/A
Software and Algorithms		
integrative genomics viewer	http://software.broadinstitute.org/software/igv/	N/A
ImageQuant	GE Health Life Science	N/A
ImageJ	https://imagej.nih.gov/ij/	N/A
Photoshop	adobe	Ver.11.0

EXPERIMENTAL MODEL AND SUBJECT DETAILS

Animals

C57BL/6J mice were used as wild-type (WT) mice. *Keratin 14* promoter-driven ectopic follistatin gene expression (*K14-Follistatin*), follistatin knockout, and *Sox21* knockout (*Sox21* KO) mice were gifted. All animal experiments were approved by the Animal Ethics Committee of Tohoku University. *Sox21* KO mice were provided with a soft diet. These mice were euthanized by cervical dislocation under isoflurane anesthesia for all experiments. All experiments were performed in accordance with the Law Concerning the Conservation and Sustainable Use of Biological Diversity through Regulations on the Use of Living Modified Organisms. All animal research and genetic recombination experiments were approved by the Tohoku University Center for Gene Research (2013DnLMO-008, 2013DnA-051).

METHOD DETAILS

SEM Analyses

The heads of 6-week-old mice were stripped of the skin and treated at 48°C in 0.3% trypsin buffer in one day. Soft tissue was removed from the skull and the molars and incisors were extracted. Teeth were embedded in epoxy resin (Oken Epok; Okenshoji Co., Ltd.). Resin was cut using a diamond disk, cleaned ultrasonically after being polished, and examined using a variable pressure scanning electron microscope

(Miniscope TM3000; Hitachi). Elemental mapping at the microstructural level was investigated by SEM with an energy dispersive X-ray spectrometry system (Quantax70; Bruker).

Micro-CT Scanner Imaging

The skulls of 6-week-old wild-type and *Sox21* KO mice were analyzed by micro-CT at the Kureha Special Laboratory (Iwaki, Japan). The mandibles were imaged and the mineralization volumes (mm^3) of enamel and dentin were calculated from the tomographically sliced sections. The tooth enamel and the dentinal quantities were determined by the density of mineralization.

Preparation of Tissue Sections and H-E Staining

Mice from E15 to post-natal 6 weeks were dissected in Dulbecco's PBS. The mandibles from the embryonic stages were fixed by 4% paraformaldehyde for 1 week. Half mandibles of P2 to post-natal 6-week-old mice were fixed and decalcified by using 2.5% paraformaldehyde and 12.5% EDTA in PBS for 2-6 weeks. The tissues were then dehydrated and embedded in paraffin. Paraffin-embedded sections (7-μm-thick) were prepared using a Leica microtome. For staining, sections were re-hydrated and stained with H-E (J. T. Baker).

In Situ Hybridization and Immunostaining

A Sox21 probe fragment comprising mouse *Sox21* mRNA [NM_177753] (nucleotides 2331–2825; 495 bp) was amplified using 5′-GATGTATAGGTGTCAGGCA-3′ and 5′-GGTCATTCACTGGTCAAG-3′ primers and cloned into pCRII-TOPO (Invitrogen). The fragment sequence was verified by sequencing. Radioactive *in situ* hybridization on 7-μm paraffin frontal and sagittal sections was carried out in accordance with standard protocols (Juuri et al., 2012). ^{35}S (Amersham)-labeled RNA probes were used for detection. Antigen was activated using Lab solution (Polysciences, Inc.) at 22°C for 15 min. Sections were incubated in 5% bovine serum albumin/PBS for 1 h prior to incubation with the primary antibody. Primary antibodies including anti-Sox2 antibody (1:100, Abcam), anti-Ameloblastin (1:50, Santa Cruz), anti-pan-keratin (1:100, Thermo Fischer Scientific), anti-Vimentin (1:100, GeneTex), anti-E-cadherin (1:20, R&D Systems), and anti-N-cadherin (1:100, GeneTex) were detected using Alexa488- or Alexa594-conjugated secondary antibodies (Thermo Fisher Scientific). Nuclei were stained with DAPI (Vector Laboratories). A fluorescence microscope (BZ-8000; Keyence) and confocal microscope (Fluoview Fv10i LIV; Olympus) were used for imaging analysis. Images were prepared using Adobe Photoshop (Adobe Systems, Inc.).

In vivo EdU staining

Edu (50 mg/kg) in PBS was intraperitoneally injected for 2 days postnatal mice. Mice were dissected after 2 h, the lower mandibles were frozen with O.C.T. Compound (Sakura Finetek Japan). Sections (20-μm-thick) were prepared using a Leica cryostat. EdU was detected in accordance with the protocol of EdU proliferation kit (Abcam). Counterstaining of Sox2 and DAPI were performed by Anti-Sox2 antibody and mounting medium with DAPI.

Cell Culture and siRNA Treatment

SF2 (rat immature dental epithelial cell line) was cultured in DMEM/F12 medium (Life Technologies) containing 10% fetal bovine serum (Life Technologies) and an antibiotic cocktail (Gibco, NY, USA) at

37°C and 5% CO_2. Mature ameloblasts differentiate from dental epithelial cells with Neurotrophin (NT)-4 (Yoshizaki et al., 2008). SF2 cells were cultured with 100 ng/mL recombinant human NT-4 (Alomone Labs) for differentiation to mature ameloblasts. For inhibition of *Sox21*, *Anapc10*, *Fzr1*, and *CDC20* expression, siRNAs were transfected into cells using Stealth RNAi™ (Thermo Fisher Scientific) with Lipofectamine™ RNAiMAX (Thermo Fisher Scientific) in accordance with the manufacturer's protocol. Cells were harvested after 48 h.

Expression Vector and Transfection

The *Sox21* open reading flame was amplified from P2 mouse incisors (5′-ATGTCCAAGCCTGTGGACCACG-3′, 5′-TAGCGCGGCAGCGTAGGC-3′). The PCR fragment was cloned into the pEF6/V5-His TOPO vector (Thermo Fisher Scientific). Full length *Sox21* sequence was verified by sequencing. SF2 cells were trypsinized and replated in a 6-well plate at a density of 1 × 10^5 cells/well. The cells were transfected with plasmid DNA using the ViaFect™ transfection reagent (Promega), which was mixed in the culture plate at a 3:1 (ViaFect™/DNA) ratio. The cells were collected for the experiments after 48 h.

RT-qPCR

Total RNA was extracted using the Quiagen RNase Kit in accordance with the manufacturer's instructions. Extracted RNA was converted into cDNA using Superscript Vilo MasterMix (Thermo Fisher Scientific). PCR was performed with the SYBR Select Master Mix (Applied Biosystems) and each specific primer (Table S2) pair using a Step One Plus Real-Time PCR system (Applied Biosystems).

Microarray Analysis

Skin and tooth germs were isolated from P1 wild-type (WT) and Sox21 KO (KO) mice. Labeled RNA samples were hybridized onto SurePrint G3 Mouse GE 8×60K microarrays (Agilent Technologies). Microarray data were analyzed using GeneSpring (Agilent Technologies). Total detected entities were filtered by signal intensity value to remove very low signal entities. Normalization was performed using a percentile shift in the analysis of signaling pathways. Therese data have been deposited in NCBI's Gene Expression Omnibus (GEO) and are accessible through GEO series accession number GSE99359, GSE99360.

ChIP-Seq

SF2 cells were transfected with the *Sox21* pCMV6-Entry vector (Origene) using ViaFect in accordance with the manufacturer's instructions. The medium was changed 24 h after transfection. After 48 h, 5 ×10^6 cells were fixed by formaldehyde and processed using the ChIP-IT Express kit (Active Motif). Cells were lysed and then sonicated to shear the chromatin. The fragmented genomic DNA (5 μg) was immunoprecipitated using the anti-DDK monoclonal antibody (Origene) overnight at 4°C. The ChIP reactions were washed and chromatin was eluted in accordance with the manufacturer's instructions. The 1 ×10^7 DNA sequencing, computational, and statistical analyses of ChIP-Seq data were performed using Active Motif. The ChIP-Seq results were visualized using integrative Genomics Viewer (IGV) software (https://www.broadinstitute.org/igv/).

Western Blotting Analysis

SF2 cells transfected with *Sox21*, *Anapc10, Fzr1*, *Cdc20*, and Scramble siRNA (Sigma-Aldrich) were plated in 6-well plates at a concentration of 1 × 10^5 cells per well and incubated for 48 h. The scramble sequence siRNA was using as the negative control. For Smad cell signaling analysis, the cells were then cultured without serum-containing medium for 2 h, followed by treatment with 1 ng/mL TGF-β1 for 0, 5, 15, 30, or 60 min at 37°C. Thereafter, the cells were washed twice with ice-cold 1 mM sodium orthovanadate (Sigma-Aldrich) in PBS, lysed with Nonidet P-40 buffer supplemented with a proteinase inhibitor mixture (Sigma-Aldrich), and centrifuged. The supernatants were then transferred to a fresh tube. For protein half-life analysis, siRNA treated cells were incubated with 300 µg/mL CHX for 0, 1, 2, and 4 h. The cells were then harvested by cell lysate buffer with a proteinase inhibitor mixture and moved to a fresh tube. The cell lysates were separated by 4-12% gradient SDS-PAGE and analyzed by western blotting. The blotted PVDF membrane was incubated with Smad3 (Cell Signaling Technology), Smad6 (Sigma-Aldrich), Smad7 (Sigma-Aldrich), or phosphor-Smad3 (Cell Signaling Technology) primary antibodies. The signals were detected using an ECL Prime kit (GE Healthcare) after treatment with a rabbit or mouse horseradish peroxidase-conjugated secondary antibody. Images were visualized using the ImageQuant LAS 4000 Mini image analysis system (GE Healthcare).

QUANTIFICATION AND STATISTICAL ANALYSIS

Relative Gene Expression Analysis by qPCR

The expression of each gene was normalized to that of *Gapdh* expression. The expression in the control sample was set to 1.0 and expression levels in the experimental samples were determined as the fold change relative to the control using the ΔΔCT method (n = 5). Statistical analysis of gene expression was performed using the Student's t test, with $p < 0.05$ considered significant.

Analysis of Cell Localization of E-cadherin and β-Actin

A line of 1500 pixels was selected from E-cadherin and β-Actin immunofluorescence images. The fluorescence intensity was measured using ImageJ software and graphed.

Data and Software Availability

In situ hybridization and immunohistochemistry photographs were merged using Adobe Photoshop. Meta data was aligned using IGV software (http://software.broadinstitute.org/software/igv/). Signal intensity was analyzed using ImageJ software (https://imagej.nih.gov/ij/). Western blotting was visualized with ImageQuant (GE Healthcare).
